# Supplementary material for: Plasma and breast milk adipokines in women across the first year postpartum and their association with maternal depressive symptoms and infant neurodevelopment: Protocol for the APPLE prospective cohort study
Source: PLoS One. 2024 Oct 25;19(10):e0310847. doi: 10.1371/journal.pone.0310847 (PMC11508165; doi:10.1371/journal.pone.0310847)
Supplement: S1 Protocol — (PDF) [file pone.0310847.s002.pdf]

PROJETO DE PESQUISA

**Título:** Adipocitocinas no plasma e leite de nutrizes no primeiro ano pós-parto: associação com sintomas depressivos e desenvolvimento cognitivo e motor do lactente

**Coordenação do projeto:** Fernanda Rebelo dos Santos

**INSTITUIÇÃO EXECUTORA: FUNDAÇÃO OSWALDO CRUZ / INSTITUTO NACIONAL DE SAÚDE DA MULHER, DA CRIANÇA, E DO ADOLESCENTE FERNANDES FIGUEIRA.**

**Resumo**

**Introdução:** O papel das adipocitocinas no sistema nervoso central tem sido foco de diversos estudos na última década. Estudos in vitro demonstram o papel da adiponectina e da leptina na estimulação da neurogênese, sendo esse um possível mecanismo pelo qual essas adipocitocinas podem agir tanto na estimulação do desenvolvimento cognitivo e motor do lactente, quanto na inibição de sintomas depressivos na puérpera. **Objetivos:** Avaliar a correlação entre adipocitocinas (adiponectina e leptina) no plasma e no leite maternos e sua associação com: o desenvolvimento cognitivo e motor infantil aos 6 e 12 meses de idade; e a saúde mental da puérpera ao longo do primeiro ano pós-parto. **Métodos:** Trata-se de uma coorte prospectiva com quatro ondas de seguimento. As puérperas e seus filhos, recém-nascidos na maternidade do IFF, serão captados na visita de acompanhamento ao Banco de Leite Humano, com até 15 dias pós-parto. O acompanhamento se dará aos 2, 6 e 12 meses pós-parto. As visitas incluirão coleta de sangue e leite maternos, aplicação da escala de Edimburgo para avaliação da saúde mental materna, aplicação da escala Bayley-III de desenvolvimento infantil, antropometria materna e infantil e avaliação da história reprodutiva, aleitamento e dados socioeconômicos e demográficos. **Resultados esperados:** A proposta pretende, primeiramente, fornecer subsídios para o esclarecimento do papel das adipocitocinas no sistema nervoso de crianças e puérperas e, consequentemente, promover a melhora no cuidado e qualidade de vida desses grupos. Adicionalmente, o projeto proverá dados sobre tipo de parto, crescimento físico infantil e variação da composição corporal materna no primeiro ano pós-parto, que são de grande relevância para a saúde pública e, quando inter-relacionados, poderão gerar resultados importantes e contribuir para a literatura existente. Ademais, o projeto tem como metas a disseminação dos resultados em eventos nacionais e internacionais e a publicação de artigos científicos, contribuindo para aumentar qualitativa e quantitativamente a produção científica nacional, além da incorporação de alunos de iniciação científica, mestrado e doutorado, contribuindo para a formação de recursos humanos altamente qualificado.

**1. Introdução**

O leite humano contém diversos nutrientes, citocinas, peptídeos, enzimas, células, imunoglobulinas, proteínas e esteróides capazes de suprir as necessidades do lactente (Ballard e Morrow 2013). Alguns desses compostos são sintetizados pelas glândulas mamárias enquanto outros são extraídos do plasma materno. O estado nutricional materno, o uso de suplementos e o consumo alimentar são alguns dos fatores que podem modificar as concentrações plasmáticas de biomarcadores e, consequentemente, a composição do leite (Codoñer-Franch et al. 2013; Bravi et al. 2016; Young et al. 2018).

Entre os biomarcadores já identificados no leite materno estão a adiponectina e a leptina, também conhecidos como biomarcadores relacionados à obesidade ou adipocitocinas, já que são secretados pelas células adiposas (Çatlı, Olgaç Dündar, e Dündar 2014). Acredita-se que essas citocinas presentes no leite materno tenham atividades hormonais em diversos tecidos do neonato enquanto seu sistema endócrino próprio não está completamente funcional, desempenhando funções no metabolismo energético e regulação da composição corporal (Savino e Liguori 2008).

Adicionalmente, estudos sugerem que as adipocitocinas podem desempenhar atividades no cérebro, como estimulação da gênese e excitabilidade neuronal do hipocampo, participando de atividades como aprendizado e memória (Harvey 2007; Liu et al. 2012; O’Malley et al. 2007; Bloemer et al. 2018; Lee et al. 2019). Há alguns anos a associação de adipocitocinas com a função cognitiva já vem sendo estudada em outros grupos populacionais, como adultos e idosos (Gorska-Ciebiada et al. 2016; Gunstad et al. 2008; Holden et al. 2009). Mais recentemente alguns estudos apresentaram dados na população infantil.

Em estudo transversal com 50 participantes realizado no Brasil, as concentrações plasmáticas de leptina se mostraram inversamente associadas ao desenvolvimento cognitivo em crianças entre 6 e 24 meses de idade (Camargos et al. 2017). Utilizando dados de duas coortes de gestantes nos Estados Unidos e Canadá, Li et al. (2019) encontraram associação entre a adiponectina no sangue do cordão umbilical e o coeficiente de inteligência e memória em crianças de 3, 5 e 8 anos de idade. Entretanto, até o presente momento não foram encontrados estudos investigando a associação das adipocitocinas no maternas e o desenvolvimento cognitivo dos lactentes durante o primeiro ano de vida.

Em se tratando dos desfechos maternos associados as concentrações de adipocitocinas em puérperas, mais esclarecimentos são necessários a respeito da saúde mental. A literatura apresenta algumas evidências de que indivíduos com agravos como depressão e ansiedade podem ter as concentrações plasmáticas de adipocitocinas alteradas (Yildiz et al. 2017; Rebelo et al. 2015; Guo et al. 2017). Porém, os dados sobre a relação entre adipocitocinas e saúde mental no período puerperal são escassos e inconclusivos (Rebelo et al. 2016).

Dessa forma, o presente projeto busca esclarecer a relação entre adipocitocinas no plasma e leite maternos, saúde mental da puérpera e desenvolvimento cognitivo e motor do lactente, partindo da hipótese de que as concentrações plasmáticas de adiponectina e leptina estão associadas ao estado de saúde mental da nutriz e correlacionadas com as concentrações das mesmas adipocitocinas no leite materno. Por sua vez, a ingestão do leite com maiores concentrações de adiponectina e menores de leptina induz à maior estimulação neuronal do lactente, propiciando um melhor desenvolvimento cognitivo e motor.

## 2. Justificativa

Esta proposta tem como objetivo produzir conhecimento científico de alta qualidade a partir da realização de estudo de caráter translacional, com desenho prospectivo ao longo do primeiro ano pós-parto. É interessante destacar que o projeto debruça-se sobre temática de alta relevância científica e com impacto sanitário importante. Tanto as exposições como os desfechos que estão sendo estudados são de grande importância para a área da saúde materna e infantil e foram pouco estudados até o momento.

O papel das adipocitocinas no sistema nervoso central tem sido foco de diversos estudos na última década. Estudos in vitro demonstram o papel da adiponectina e da leptina na estimulação da neurogênese, regulando a proliferação de células do hipocampo de forma tempo e dose dependente (Zhang et al. 2011; Garza et al. 2008). Portanto, o estímulo à neurogênese é um possível mecanismo pelo qual essas adipocitocinas podem agir tanto na estimulação do desenvolvimento cognitivo e motor do lactente, quanto na inibição de sintomas depressivos na puérpera.

O período em que o crescimento cerebral atinge maior velocidade e apresenta maior plasticidade ocorre no último trimestre da gravidez e nos dois primeiros anos de vida. Dessa forma, os primeiros mil dias de vida, da concepção aos 2 anos de idade, são apontados como cruciais na trajetória de desenvolvimento, sendo determinantes do estado de saúde futuro, e estão associados a maior capacidade cognitiva e de aprendizado durante a vida adulta (Grantham-McGregor et al. 2007). Dessa forma, a identificação de fatores que possam estar associados ao neurodesenvolvimento no primeiro ano de vida, considerado uma janela de oportunidades para intervenções, é de grande relevância para contribuir na saúde e qualidade de vida da população.

Já a depressão pós-parto é especialmente problemática por afetar não somente a saúde da mulher, como também interferir negativamente na saúde da criança e ter o tratamento medicamentoso dificultado, pois existe a transmissão do fármaco para a criança, através do leite materno (Gross et al. 2013; Jensen, Dumontheil, e Barker 2013; Lanza di Scalea e Wisner 2009; Sit et al. 2011). Mães com depressão costumam ter a saúde física comprometida e comportamentos de alto risco, como abuso de álcool e outras substâncias. Essas mulheres são menos propensas a cuidar adequadamente de suas próprias necessidades e de seus filhos e têm maior resistência a procurar e receber cuidados pós-natal ou aderir às prescrições médicas (Alder et al. 2007). Além disso, o suicídio, cujo principal fator de risco é a depressão, é a principal causa de morte materna em países desenvolvidos (Oates 2003).

Visto a escassez de estudos com este objeto, a presente proposta se destaca pelo seu potencial inovador e capacidade de gerar evidências capazes de auxiliar no entendimento e na prevenção de problemas de desenvolvimento na infância e ao longo do ciclo da vida, além de auxiliar na busca de novas estratégias para prevenir e tratar a depressão pós-parto.

# **3.** Objetivos

## *3.1 Objetivo geral*

Avaliar a correlação entre adipocitocinas (adiponectina e leptina) no plasma e no leite maternos e sua associação com: o desenvolvimento cognitivo e motor infantil aos 6 e 12 meses de idade; e a saúde mental da puérpera ao longo do primeiro ano pós-parto.

## *3.2 Objetivos específicos*

- Descrever e correlacionar as concentrações de adipocitocinas no plasma e leite maternos no primeiro ano pós-parto, de acordo com variáveis selecionadas, incluindo a variação ponderal materna e o tipo de parto;
- Descrever o desenvolvimento cognitivo e o crescimento físico infantil no primeiro ano de vida de acordo com variáveis maternas selecionadas;
- Descrever a ocorrência de sintomas depressivos nas puérperas ao longo do primeiro ano pós-parto de acordo com variáveis maternas selecionadas;
- Avaliar a associação entre a saúde mental das lactantes e as concentrações de adiponectina e leptina maternas;
- Avaliar a associação entre saúde mental das puérperas e o crescimento físico e desenvolvimento cognitivo e motor dos lactentes no primeiro ano de vida;
- Avaliar a associação entre as adipocitocinas maternas e o crescimento físico e desenvolvimento cognitivo e motor dos lactentes no primeiro ano de vida;
- Avaliar o papel mediador das adipocitocinas no leite materno e da saúde mental da puérpera na relação entre adipocitocinas no plasma materno e o crescimento físico e desenvolvimento cognitivo e motor do lactente no primeiro ano de vida.
- Avaliar a associação entre qualidade do sono e vínculo mãe-bebê com a saúde mental materna;
- Avaliar a associação do consumo de ultraprocessados com a concentração de adipocitocinas no plasma e leite das lactantes;
- Avaliar a validade do acompanhamento dos marcos do desenvolvimento da Caderneta de Saúde da Criança para identificação de déficits de desenvolvimento infantil.
- Descrever e correlacionar as concentrações de biomarcadores inflamatórios e metabólitos de vitamina D no plasma e leite maternos no primeiro ano pós-parto,

de acordo com variáveis selecionadas, incluindo a variação ponderal materna, o tipo de parto e o consumo de ultraprocessados;

- Avaliar a associação as concentrações de biomarcadores inflamatórios e metabólitos de vitamina D no leite e sangue maternos e sintomas depressivos em puérperas;
- Avaliar a associação entre as concentrações de biomarcadores inflamatórios e metabólitos de vitamina D no leite e sangue maternos e o desenvolvimento cognitivo e motor aos 6 e 12 meses;

## 4. Metas

i. Fornecer subsídios para o esclarecimento do papel das adipocitocinas no sistema nervoso de crianças e puérperas e, consequentemente, promover a melhora no cuidado e qualidade de vida desses grupos;
ii. Prover dados sobre tipo de parto, crescimento físico infantil e variação ponderal e da composição corporal materna no primeiro ano pós-parto. Esses dados, de grande relevância para a saúde pública, quando inter-relacionados, poderão gerar resultados importantes e contribuir para a literatura existente.
iii. Disseminar os resultados obtidos em seminários locais, congressos nacionais e internacionais e por meio da publicação de artigos científicos, contribuindo para aumentar qualitativa e quantitativamente a produção científica nacional;
iv. Potencializar a formação de recursos humanos para a área de pesquisa e ensino, por meio da interlocução, em caráter multidisciplinar, da equipe de pesquisadores e alunos de doutorado, mestrado e iniciação científica que serão incorporados a equipe;
v. Estimular a interação entre os departamentos do Instituto Fernandes Figueira (IFF) envolvidos na presente proposta, promovendo a troca de conhecimentos, incitando o desenvolvimento de projetos futuros em caráter multidisciplinar e aprimorando a assistência prestada aos usuários da Instituição.

## 5. Métodos

Para facilitar a comunicação, o título da pesquisa “Adipocitocinas no plasma e leite de nutrizes no primeiro ano pós-parto: associação com sintomas depressivos e desenvolvimento cognitivo e motor do lactente” será abreviado com a sigla APPLE.

Trata-se de uma coorte prospectiva com quatro ondas de seguimento: puerpério imediato (até 15 dias pós-parto), 2º mês pós-parto, 6º mês pós-parto e 12º mês pós-parto. Serão convidadas a participar do estudo lactantes adultas e seus filhos que estiverem no período de até 15 dias pós-parto. A captação das participantes será realizada em duas frentes: (1) divulgação no alojamento conjunto do IFF, com posterior busca ativa das puérperas elegíveis que retornarem para o acompanhamento de puericultura no Banco de Leite Humano (BLH) do IFF; (2) divulgação para contatos pessoais da equipe do projeto em tática bola de neve, onde as interessadas irão entrar em contato por email ou telefone para avaliação da elegibilidade e agendamento da primeira avaliação.

O acompanhamento se dará no BLH (coleta de leite), no serviço de pediatria (avaliação de desenvolvimento infantil) e no Laboratório de Nutrição e Metabolismo (avaliação da composição corporal, coleta de sangue e aplicação dos questionários). O detalhamento das variáveis obtidas em cada onda de seguimento do estudo pode ser observado na **Figura 1**. O recrutamento seguirá os critérios de elegibilidade descritos no **Quadro 1**.

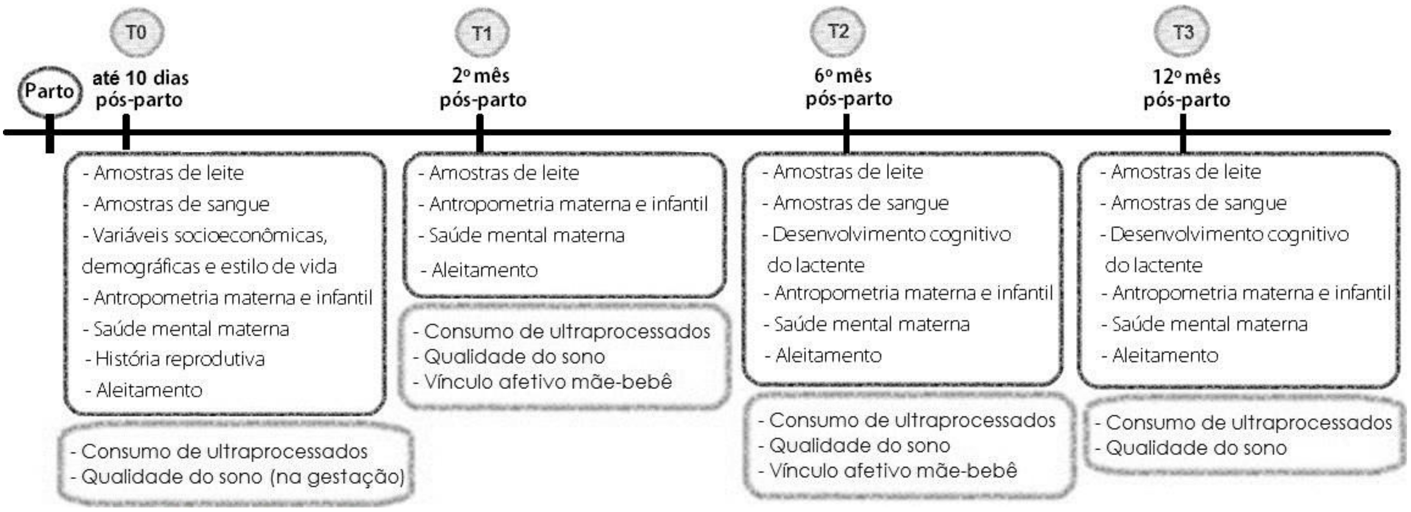


**Figura 1.** Dados obtidos em cada onda de seguimento da coorte.

**Quadro 1.** Critérios de elegibilidade.

| <u>Critérios de inclusão</u> → **Serão selecionadas lactantes que atenderem aos seguintes critérios de elegibilidade no momento de recrutamento:** | <u>Critérios de exclusão</u> → **Deverão ser excluídas as lactantes :** |
|---|---|
| 1. Estar com até 15 dias pós-parto; | 1. Cujo recém-nascido teve parto prematuro; |
| 2. Ter entre 20 e 45 anos de idade; | 2. Cujo recém-nascido apresente alguma patologia ou peso não adequado para idade gestacional (<p5 ou >p95, Villar et al. 2014); |
| 3. Estar livre de doenças crônicas não transmissíveis como hipertensão arterial e diabetes, exceto obesidade; | 3. Em uso de medicação antidepressiva ou outra medicação psiquiátrica. |
| 4. Estar livre de doenças infecciosas, como HIV; | 4. Que apresentaram gestação gemelar; |
| 5. Estar realizando aleitamento exclusivo, definido como a oferta exclusiva de leite materno, sem chás ou água, nos 5 dias que precederem a entrada no estudo. | 5. Cujo recém-nascido apresente diagnóstico de qualquer agravo ou malformação que sabidamente cause déficits motores e/ou cognitivos; |
| 6. Residir na cidade do Rio de Janeiro; | 6. Que tiveram sorologia positiva na gestação para Sífilis, Rubéola, Toxoplasmose, CMV ou Zika; |
| 7. Recém-nascido com Apgar ≥7 no quinto minuto; | 7. Cujo recém-nascido tenha ficado internado em UTI neonatal; |
| 8. Domínio da língua portuguesa. | 9. Mulheres que tiveram gestação para útero de substituição/ cessão temporária de útero ("barriga solidária") ou que vão entregar o bebê para adoção. |

*5.1 Variáveis do estudo*

- Amostras de sangue e leite materno

O leite humano (mínimo 10 ml e máximo 20 ml) será extraído manualmente, sendo uma amostra recolhida no início e outra no final da amamentação, pela lactante. Estas amostras serão armazenadas e transportadas, sob refrigeração, em um prazo máximo de 2 horas ao local de realização das análises e homogeneizadas de forma ultrassônica (20 Hz durante 1,5 segundos/ml). Posteriormente, serão separadas em alíquotas e armazenadas a -20ºC até o processamento e análise. As amostras serão obtidas em todas as visitas de acompanhamento ou até que a puérpera não tenha mais leite.

Amostras de sangue serão extraídas em nas visitas T0, T2 e T3 em tubo de coleta contendo EDTA para determinação da concentração de adipocitocinas por meio de kits comerciais de ELISA. As amostras serão centrifugadas (5.000 rpm/5 minutos) e o plasma será separado em criotubos e armazenado em freezer à -20°C até a data de realização das análises.

Além das adipocitocinas, também serão dosados biomarcadores inflamatórios e metabólitos de vitamina D. Os biomarcadores inflamatórios, como oxilipinas e frações de ácidos graxos, serão analisados por cromatografia liquida de alta resolução acoplada a espectrometria de massas (HPLC-MS/MS)(Durand et al. 2001; 2002). Os metabólitos de Vitamina D serão analisados por HPLC-MS/MS (Agilent® 1260 Infinity), com uma coluna Lux® celulose F5 (Phenomenex), acoplado espectrômetro de massas – AB Sciex QTrap® 5500, operando no modo positivo (Satoh et al. 2016).

- Desenvolvimento cognitivo e motor do lactente

O desenvolvimento cognitivo e motor será avaliado por meio das escalas Bayley de Desenvolvimento Infantil (BSID-III) no 6º e 12º mês após o parto (margem de 15 dias para mais ou para menos a contar da data exata). Esse instrumento é destinado à avaliação de crianças de 1-42 meses e composta pelas seguintes escalas: (i) Escala Cognitiva, que avalia o desenvolvimento sensório-motor, a exploração e manipulação, formação de conceitos e memória; (ii) Escala de Linguagem, composta por itens de comunicação receptiva e expressiva, formando dois sub-testes distintos; (iii) Escala Motora, que avalia habilidades motoras em geral e motricidade fina; (iv) Escala Sócio-emocional, que identifica os principais marcos do desenvolvimento social e emocional em determinadas idades; (v) Escala de Comportamento Adaptativo, que acessa habilidades funcionais do cotidiano da criança e considera a comunicação, vida em casa, saúde, segurança e lazer.

Adicionalmente, serão avaliados os marcos de desenvolvimento presentes na Caderneta de Saúde da Criança. Essa avaliação é realizada de forma rotineira na clínica pediátrica e verificar sua validade a partir com a comparação com os resultados da BSID-III (padrão ouro) será mais um dado importante gerado pelo presente estudo.

O resultado da avaliação será comunicado ao pediatra responsável, para que as devidas providências sejam tomadas, permitindo que haja o encaminhamento para um especialista caso necessário.

- Saúde mental materna

A saúde mental materna será monitorada em todas as ondas de seguimento: até 15 dias, 2, 6 e 12 meses pós-parto. Na primeira consulta serão coletados dados sobre o histórico de depressão. Será utilizada uma versão validada da Escala de Depressão Pós-parto de Edinburgh (EPDS). A escala foi desenvolvida por Cox et al. (Cox, Holden, e Sagovsky 1987) e traduzida e validada para o português por Santos et al. (Santos et al. 2007) e consiste em um instrumento de auto registro composto por 10 enunciados, com opções pontuadas de 0 à 3 de acordo com a presença e a intensidade do sintoma depressivo (Anexo 1).

Vários são os estudos que citam e utilizam a EPDS por ser um questionário de fácil aplicação e entendimento para a mulher, além de se apresentar bastante eficiente na identificação da depressão pós-parto (Boyce e Hickey 2005; Buist, Westley, e Hill 1999; Hibbeln 2002; Otto, de Groot, e Hornstra 2003). A participante será incentivada a completar sozinha as perguntas do questionário, mas contará com a ajuda de um entrevistador treinado para esclarecer qualquer dúvida ou auxiliá-la na leitura das perguntas e preenchimento das respostas caso ela não consiga executá-lo de forma independente.

Adicionalmente, haverá a avaliação da depressão por meio do Inventário de Depressão de Beck-II (BDI-II) aos 6 e 12 meses pós-parto. Isso porque a validação da versão brasileira da EPDS foi realizada apenas com mulheres com até 3 meses pós-parto. O BDI-II é um instrumento utilizado amplamente em todo o mundo e validado para a população brasileira(Gomes-Oliveira et al. 2012). A aplicação será realizada por psicólogo treinado.

O resultado da avaliação será comunicado ao ginecologista responsável, para que as devidas providências inerentes ao tratamento adequado sejam tomadas, permitindo que haja o encaminhamento para um especialista caso necessário.

- Variáveis socioeconômicas, demográficas, estilo de vida e história reprodutiva

Será aplicado um questionário estruturado para determinar as características socioeconômicas e demográficas maternas, bem como os dados de estilo de vida e dados reprodutivos que figuram na história da nutriz: idade, estado civil, número de filhos, ocupação, nível de escolaridade, local de moradia, tipo de moradia, tabagismo, etilismo e sexo do recém-nascido; número de partos, data do último parto, tipo de parto (especificando o motivo para o parto cirúrgico, quando for o caso) e duração da gestação.

- Antropometria materna e infantil

Na primeira consulta serão coletados dados sobre o peso pré-gestacional da mulher. Para isso será dado preferência ao peso aferido até a 13ª semana gestacional registrado na caderneta de acompanhamento da gestante. Caso esse registro seja inexistente será registrado o peso relatado. Já para o recém-nascido serão coletados os seguintes dados contidos na Caderneta de Saúde da Criança: peso ao nascer, comprimento ao nascimento e perímetro cefálico ao nascimento.

Para a avaliação da composição corporal das puérperas e de bebês com até 8kg será utilizado o método de pletismografia por deslocamento de ar que avalia o volume e a densidade corporal através da mensuração do volume de ar deslocado pelo corpo dentro de uma câmera fechada. É um método relativamente recente, rápido, seguro, confortável (Fields, Higgins, e Hunter 2004). Esta técnica é baseada na lei de Boyle que se fundamenta na relação inversa entre pressão e volume para determinar a composição corporal. Definido esse volume, é possível aplicar os princípios da densitometria para determinar a composição corporal por meio do cálculo da densidade corporal (Mello et al. 2005). O pletismógráfo, também chamado de BOD POD para adultos ou PEA POD para bebês, é acoplado a um computador que determina as variações no volume de ar e de pressão no interior da câmara vazia e ocupada. Esse equipamento realiza ajustes para variáveis pulmonares necessárias na estimativa do volume corporal (Guedes, 1998). O equipamento também fornecerá o peso da mulher e da criança a cada entrevista.

A estatura será avaliada de forma direta por meio de um estadiômetro padrão de alumínio (SN4010, Sanny®) com precisão de um milímetro durante a primeira onda de seguimento.

Quando a criança superar o peso de 8kg (capacidade máxima do PEA POD), a aferição do peso da criança será realizada em balança pediátrica digital com capacidade máxima de 20kg. Estima-se que a avaliação da composição corporal infantil só será possível até os 6 meses. O comprimento da criança será aferido em infantômetro, com a criança deitada, com os pés apoiados na parte fixa do instrumento. A parte móvel será adequadamente posicionada sobre a cabeça da criança. A medida será realizada em duplicata, sendo utilizada a média das aferições. Também será aferido o perímetro cefálico/circunferência "frontoccipital" da criança, utilizando-se fita métrica extensível, correspondendo ao perímetro cefálico máximo.

- Aleitamento

Será aplicado um questionário sobre aleitamento materno em todas as ondas de seguimento. A frequência de consumo de leite materno será estimada por meio de recordatório de 24 horas. O instrumento utilizado será adaptado da II Pesquisa de Prevalência de Aleitamento Materno nas Capitais Brasileiras e Distrito Federal, do Ministério da Saúde (Venancio et al. 2010).

- Consumo alimentar de ultraprocessados

O consumo alimentar de ultraprocessados será avaliado em todas as ondas de seguimento utilizando-se o Escore Nova (Costa et al. 2021). Trata-se de um questionário auto-aplicável com duração em torno de três minutos que mostrou-se com bom desempenho para a estimação da participação de ultraprocessados na dieta do brasileiro. O questionário será enviado por meio eletrônico (e-mail ou whatsapp) uma semana antes das consultas presenciais (exceto para primeira consulta). Para as mulheres que não responderem o questionário eletrônico, este será aplicado durante a consulta presencial.

- Qualidade do sono

A qualidade do sono será avaliada por meio do Índice de qualidade de sono de Pittsburgh traduzido e validado para a população brasileira (PSQI-BR)(Bertolazi et al. 2011). Na primeira onda de seguimento (até 15 dias pós-parto) as perguntas irão se referir ao período gestacional. Nas demais ondas será requisitado que a mulher responda com base no período atual. O questionário será enviado por meio eletrônico (e-mail ou whatsapp) uma semana antes das consultas presenciais (exceto para primeira consulta). Para as mulheres que não responderem o questionário eletrônico, este será aplicado durante a consulta presencial.

- Vínculo afetivo mãe-bebê

O vínculo afetivo mãe-bebê será avaliado na segunda e terceira ondas de seguimento (T1 e T2), por meio da versão traduzida do *Postpartum Bonding Questionnaire* (PBQ)(Baldisserotto et al. 2018). O questionário será enviado por meio eletrônico (e-mail ou whatsapp) uma semana antes das consultas presenciais (exceto para primeira consulta). Para as mulheres que não responderem o questionário eletrônico, este será aplicado durante a consulta presencial.

*5.2 Assistência aos participantes*

A ocorrência de sintomas depressivos nas mulheres é o resultado crítico que se espera ser mais recorrente durante o acompanhamento do projeto. As mulheres que forem classificadas com sintomas depressivos pela escala EPDS ou tiverem o diagnóstico de depressão pelo BDI-II serão encaminhadas para atendimento na Coordenação de Saúde Mental do IFF (COJ), caso tenham matrícula no IFF. As participantes que não tiverem matrícula no IFF serão encaminhadas para atendimento no ambulatório do Instituto de Psiquiatria da UFRJ (IPUB).

No caso de outros resultados críticos, a equipe do projeto se responsabilizará para que os participantes recebam o atendimento adequado. Ressalta-se que a equipe é multiprofissional, composta por profissionais das áreas de pediatria, psiquiatria, neonatologia, psicologia, nutrição e enfermagem.

*5.3 Tamanho amostral*

Para avaliar as diferenças de adipocitocinas no leite humano de acordo com o desenvolvimento infantil, utilizaremos como referência o escore global de desenvolvimento cognitivo no domínio comunicação. Estudo prévio aceita que o desvio padrão do escore é de 13,6. (Filgueiras et al. 2013). Consideraremos significativa uma diferença de 3 pontos. Aceitamos um risco de 0,05 e desejamos um poder estatístico de 95% para detectar diferenças, se existirem.

Aplicando a fórmula de determinação do tamanho da amostra com base em uma estimativa de média populacional ($n=[Z\alpha/2.\delta/E]^2$) teremos N = 79 (Bolfarine e Bussab 2005). Se estimarmos uma perda de seguimento de aproximadamente 20%, o N amostral será de 95 pares de mães e filhos.

*5.4 Digitação dos dados e análise estatística*

As informações coletadas serão inseridas no banco de dados por meio de dupla digitação no software RedCap, em máscaras de entrada de dados especificamente desenvolvidas para o estudo.

As análises estatísticas serão realizadas com o pacote estatístico STATA. Primeiramente será realizada uma avaliação dos dados por meio de análise de dispersão. A normalidade dos dados será investigada utilizando-se o teste de Kolmogorov-Smirnoff e as variáveis não normais serão submetidas a uma transformação logarítmica para sua análise posterior por testes paramétricos. As proporções de variáveis categóricas serão comparadas por meio de teste de chi-quadrado e as diferenças entre médias de variáveis contínuas serão testadas com T-Student. Para a correlação entre variáveis contínuas será utilizado o coeficiente de correlação de Pearson (por exemplo, correlação entre adipocitocinas no plasma e no leite).

As principais variáveis (concentrações de adipocitocinas, escore de desenvolvimento e sintomas depressivos) serão trabalhadas na forma contínua. Para avaliar a associação entre elas será utilizado um modelo longitudinal de efeitos mistos (LME, acrônimo do inglês Linear Mixed Effects), ajustado por fatores de confundimento. O LME é um modelo capaz de capturar mudanças entre e intra indivíduos, acomodar covariáveis tempo-dependentes e independentes, levar em conta a correlação entre medidas repetidas no mesmo indivíduo e permite intervalos de tempo desbalanceados. O

tempo pós-parto será incluído como variável de efeito fixo e aleatório. Caso a resposta seja descrita por uma função não linear, um termo quadrático será inserido no modelo.

Para determinar as variáveis de ajuste do modelo, será utilizado o gráfico acíclico direcionado (DAG, do acrônimo em inglês *Direct Acyclic Graph*) construído usando o software online DAGitty (Textor, Hardt, e Knüppel 2011). O objetivo do DAG é melhorar nosso entendimento da associação entre os exposição e resultado e todos os possíveis fatores de confusão. Essa abordagem permite identificar o ajuste mínimo suficiente definido para estimar o efeito direto das concentrações plasmáticas de adiponectina e odesenvolvimento cognitivo e motor do lactente. Uma primeira versão do DAG pode ser observada na **Figura 2**.

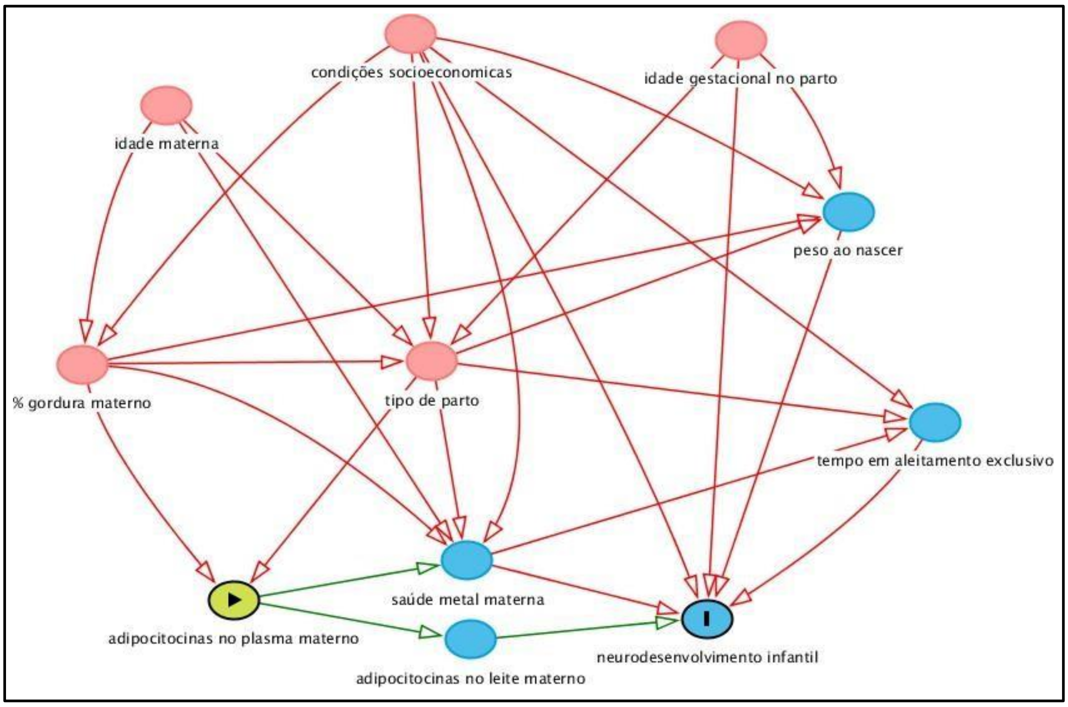


**Figura 2.** Gráfico acíclico direcionado da relação entre adipocitocinas no plasma materno e neurodesenvolvimento infantil. Ajuste mínimo necessário: percentual de gordura materno e tipo de parto.

*5.5 Questões éticas*

O projeto está em acordo com as Resoluções nº 466, de 12 de dezembro de 2012 e nº 510, de 7 de abril de 2016. A participação na pesquisa estará condicionada à assinatura de duas vias do termo de consentimento, que será obtido de forma livre e espontânea, após terem sido feitos todos os esclarecimentos pertinentes ao presente estudo.

## 6. Produtos esperados, impactos do projeto e viabilidade de aplicação do conhecimento produzido.

A proposta pretende, primeiramente, promover avanços no entendimento das relações entre adipocitocinas do plasma e leite materno, desenvolvimento cognitivo e motor do lactente e saúde mental materna. Adicionalmente, o projeto proverá dados sobre tipo de parto, crescimento físico infantil e variação ponderal materna no primeiro ano pós-parto. Esses dados, de grande relevância para a saúde pública, quando inter- relacionados, poderão gerar resultados importantes e contribuir para a literatura existente.

Alguns estudos in vitro já sugerem que as adipocitocinas apresentam funções na regulação neuronal. Ao que tudo indica, esse será o primeiro estudo epidemiológico a prover evidências sobre a associação de adipocitocinas maternas com o desenvolvimento cognitivo do lactente e com a saúde mental materna no primeiro ano pós-parto. Neste momento, um estudo observacional que mostre essas possíveis associações irá gerar evidências, levantar hipóteses e incitar mais estudos com esse tema.

Espera-se que os resultados sirvam como estímulo ao aleitamento materno, além de auxiliar no entendimento e na prevenção de problemas de desenvolvimento na infância e ao longo do ciclo da vida. Futuramente, a investigação dos fatores associados às concentrações de adipocitocinas no leite materno e das intervenções que podem modular essas concentrações poderão suscitar novas recomendações às puérperas, gerando impacto na capacidade de aprendizagem infantil e, consequentemente, melhora no seu bem-estar social, emocional e cognitivo.

Adicionalmente, considerando a necessidade de mais evidências sobre a associação entre adiponectina e depressão, a falta de estudos sobre o tópico em puérperas e o impacto da depressão na saúde materna e infantil, a avaliação da associação entre

adipocitocinas e saúde mental em puérperas é um caminho para busca de novas estratégias para prevenir e tratar depressão pós-parto.

Sendo assim, o projeto colabora com a produção e disseminação de conhecimentos, contribui na promoção da saúde da mulher e da criança e qualidade de vida da população brasileira e oferece subsídios à execução e formulação de políticas públicas nacionais, encontrando-se articulado com o plano institucional da Fiocruz e do IFF.

*6.1 Produtos esperados*

i. Formação de recursos humanos: 1 trabalho de iniciação científica; 2 dissertações de mestrado; 1 tese de doutorado;
ii. Produção e disseminação do conhecimento: 4 artigos publicados em revistas internacionais; 4 resumos apresentados em congressos nacionais e internacionais;

**7. Metas (definição em termos quantitativos) e Critérios de avaliação**

| **Metas** | **Critérios de Avaliação** |
|---|---|
| Inclusão de 95 participantes | Assinatura do Termo de Consentimento Livre e Esclarecido (TCLE) – Durante a execução do estudo. |
| Contribuição para 2 dissertações de mestrado e 1 tese de doutorado. | Publicação no Repositório Institucional da Fiocruz. Disponível em: https://www.arca.fiocruz.br |
| Elaboração de resumo executivo do impacto do projeto. | Produção de relatório final – Ao final do estudo. |
| Envio de quatro artigos científicos para ampla difusão na sociedade (nacional e internacional). | 4 artigos em execução ao final dos 24 meses de projeto |

| | |
|---|---|
| Envio de resultados para ao menos dois eventos científicos. | 11º Congresso Brasileiro de Epidemiologia, Fortaleza/ CE, 2020<br>DOHaD Society World Congress, Vancouver/ Canadá, 2022 |

**8. Localidade**

As nutrizes e seus filhos recém-nascidos na maternidade do IFF serão captados nas dependências do Banco de Leite Humano. O acompanhamento se dará no Banco de Leite Humano (coleta de leite), no serviço de pediatria (avaliação de desenvolvimento infantil) e no Laboratório de Nutrição e Metabolismo (avaliação da composição corporal, coleta de sangue e aplicação dos questionários).

**9. Público alvo do Projeto, beneficiários diretos e indiretos e prescrever ações de interação entre pesquisadores e público alvo**

O público alvo desse projeto serão as puérperas e seus filhos nascidos na maternidade do IFF. Visando potencializar o recrutamento de participantes, serão distribuídos folders no alojamento conjunto (beira leito), para as pacientes que se enquadrarem nos critérios de elegibilidade. Os folders irão ressaltar a possibilidade de realização do acompanhamento da composição corporal e do desenvolvimento do bebê ao longo do próximo ano e convidá-las a conversar com a equipe durante a consulta de puericultura no BLH (**Anexo 2**).

Espera-se que os resultados sirvam como estímulo ao aleitamento materno, além de auxiliar no entendimento e na prevenção de problemas de desenvolvimento na infância e ao longo do ciclo da vida. Futuramente, a investigação dos fatores associados às concentrações de adipocitocinas no leite materno e das intervenções que podem modular essas concentrações poderão suscitar novas recomendações às puérperas, gerando impacto na capacidade de aprendizagem infantil e, consequentemente, melhora no seu bem-estar social, emocional e cognitivo.

Adicionalmente, considerando a necessidade de mais evidências sobre a associação entre adiponectina e depressão, a falta de estudos sobre o tópico em puérperas

e o impacto da depressão na saúde materna e infantil, a avaliação da associação entre adipocitocinas e saúde mental em puérperas é um caminho para busca de novas estratégias para prevenir e tratar depressão pós-parto.

A interação entre pesquisadores e público alvo se dará durante todo o decorrer do projeto por meio da leitura do Termo de Consentimento Livre e Esclarecido, a realização das entrevistas e exames e sempre que as participantes necessitarem explicações adicionais. Além disso, a divulgação dos resultados do presente estudo será realizada por meio workshops para às puérperas usuárias do Banco de Leite Humano, como também para os funcionários envolvidos.

## 10. Detalhar o programa de formação continuada, de treinamento e capacitação de recursos humanos qualificados

Todos os integrantes do presente projeto serão treinados e capacitados para a realização das entrevistas e aplicação da escala EPDS.

- Entrevista: será realizado um treinamento prático com o grupo de pesquisadores que farão as entrevistas para observar como os mesmos estão realizando as perguntas e anotações no questionário a fim de evitar erros na indagação das questões com o objetivo principal de evitar a introdução de viés de informação.
- Avaliação da composição corporal: uma bolsista de pesquisa do IFF, treinada e capacitada no uso do BOD POD e PEA POD está responsável por essa avaliação. Se necessário, outros membros da equipe serão devidamente treinados.
- Avaliação do neurodesenvolvimento do recém-nascido: fará parte da equipe uma psicóloga treinada e capacitada para a utilização da escala Bayley-III.
- Após todos estes treinamentos, será realizado estudo piloto para avaliar novamente os questionários, o fluxograma da pesquisa, a performance dos entrevistadores para o uso dos instrumentos.

## 11. Orçamento justificado

Duração do projeto: 24 meses

Tabela 1. Orçamento

| Descrição | Quantidade | Valor unitário (R$) | Valor total (R$) |
|---|---|---|---|
| **CUSTEIO** | | | |
| **Material de escritório** (papéis, pastas, cartuchos, canetas, cadernos etc) | 1 | 2.000,00 | 2.000,00 |
| **Material para dosagem das citocinas** | | | |
| KIT ELISA para dosagem de adiponectina | 5 | 2.670,00 | 13.350,00 |
| KIT ELISA para dosagem de leptina | 5 | 2.670,00 | 13.350,00 |
| **Material para coleta das amostras** | | | |
| Criotubos para armazenamento de amostras (pacote com 1000) | 2 | 318,00 | 636,00 |
| Caixas para armazenamento de criotubos | 14 | 7,00 | 98,00 |
| Coletor universal para coleta de leite (pacote c/ 10) | 38 | 5,50 | 209,00 |
| Agulhas descartáveis (caixa com 100) | 3 | 30,00 | 90,00 |
| Seringas descartáveis (caixa com 100) | 3 | 70,00 | 210,00 |
| Luvas descartáveis (caixa com 100) | 6 | 24,00 | 144,00 |
| Algodão em rolo | 5 | 15,00 | 75,00 |
| Tubo EDTA para coleta (caixa com 100) | 3 | 47,00 | 141,00 |
| Garrote | 3 | 16,00 | 48,00 |
| SCALP (caixa com 100) | 3 | 27,00 | 81,00 |
| **Avaliação do desenvolvimento infantil** | | | |
| Formulários da Escala Bayley-III | 190 | 5,00 | 950,00 |
| **Publicação de artigos** | | | |
| Revisões e traduções | 1 | 1.000,00 | 1.000,00 |
| **Ressarcimento das participantes** | 380 | 40,00 | 15.200,00 |
| **Subtotal 1 (Custeio)** | | | 47.582,00 |
| **BOLSAS** | | | |
| **Nível médio** | 18 | 1.200,00 | 21.600,00 |
| **Nível superior** | 12 | 3.000,00 | 36.000,00 |
| **Subtotal 2 (Bolsas)** | | | 57.600,00 |
| **TOTAL** | | | |

O orçamento está prevendo gasto com material de escritório para impressão de formulários, armazenamento de documentos e registros de atividades. Está sendo requisitado verba para comprar os materiais necessários para a coleta de sangue e para a

dosagem das citocinas serão comprados kits comerciais de ELISA. Todo material de laboratório necessário para essa análise está disponível na Instituição.

Foi orçado o custo de impressão dos formulários da Escala Bayley-III, que será utilizada para avaliação do desenvolvimento infantil.

Estão sendo requisitadas duas bolsas. Uma será destinada a um profissional de nível médio que fará as coletas de sangue. A segunda será destinada a um profissional de nível superior, de preferência com experiência na área de pesquisa, para fazer a coordenação de campo.

Será reservado R$15.200 reais para ressarcimento de despesas decorrentes da pesquisa para a participante e seu acompanhante. O ressarcimento se dará em todas as avaliações em que a participante vier ao IFF exclusivamente para o projeto. Dessa forma, as pacientes externas ao IFF receberão ressarcimento em todas as avaliações.

A infraestrutura básica (sala, mobiliário mínimo composto por mesas e cadeiras, e armário) e equipamentos serão providos pelo Instituto Nacional da Saúde da Mulher, da Criança e do Adolescente, estando disponível para uso: Kit de manipuláveis Bayley- III, BOD-POD, computadores e impressoras.

**Cronograma**

A coleta de dados terá início em setembro de 2022, com duração estimada de 27 meses, sendo 15 de captação de participantes.

**Tabela 2.** Cronograma

| ATIVIDADES | Período | |
|---|---|---|
| | Início | Término |
| **Revisão da literatura** | Janeiro 2022 | Julho 2025 |
| **Treinamento dos entrevistadores** | Janeiro 2022 | Agosto 2022 |
| **Inclusão dos participantes** | Setembro 2022 | Dezembro 2023 |
| **Coleta de dados** | Setembro 2022 | Dezembro 2024 |
| **Entrada, monitoramento e análises preliminares** | Setembro 2022 | Dezembro 2024 |
| **Análises laboratoriais das amostras de sangue e leite (T0)** | Janeiro 2024 | Fevereiro 2024 |
| **Análises laboratoriais das amostras de leite (T1)** | Março 2024 | Abril 2024 |
| **Análises laboratoriais das amostras de sangue e leite (T2)** | Julho 2024 | Agosto 2024 |
| **Análises laboratoriais das amostras de sangue e leite (T3)** | Janeiro 2025 | Fevereiro 2025 |
| **Sessões científicas para discussão dos resultados** | Março 2023 | Março 2025 |
| **Elaboração de artigos científicos** | Dezembro 2023 | Julho 2025 |
| **Análise dos dados** | Dezembro 2023 | Julho 2025 |
| **Elaboração do relatório final e prestação de contas** | Julho 2024 | Julho 2025 |
| **Retorno dos resultados de composição corporal e desenvolvimento infantil aos participantes** | Setembro 2022 | Dezembro 2024 |
| **Devolutiva do relatório de resultados do estudo aos participantes** | Julho 2024 | Julho 2025 |

**Referências Bibliográficas**


Alder, Judith, Nadine Fink, Johannes Bitzer, Irene Hösli, e Wolfgang Holzgreve. 2007. "Depression and Anxiety during Pregnancy: A Risk Factor for Obstetric, Fetal and Neonatal Outcome? A Critical Review of the Literature". *The Journal of Maternal-Fetal & Neonatal Medicine: The Official Journal of the European Association of Perinatal Medicine, the Federation of Asia and Oceania Perinatal Societies, the International Society of Perinatal Obstetricians* 20 (3): 189–209. https://doi.org/10.1080/14767050701209560.

Baldisserotto, Márcia Leonardi, Mariza Miranda Theme-Filha, Rosane Harter Griep, John Oates, Joel Renó Junior, e Juliana Pires Cavalsan. 2018. "Transcultural Adaptation to the Brazilian Portuguese of the *Postpartum Bonding Questionnaire* for Assessing the Postpartum Bond between Mother and Baby". *Cadernos de Saúde Pública* 34 (agosto). https://doi.org/10.1590/0102-311X00170717.

Ballard, Olivia, e Ardythe L. Morrow. 2013. "Human Milk Composition: Nutrients and Bioactive Factors". *Pediatric clinics of North America* 60 (1): 49–74. https://doi.org/10.1016/j.pcl.2012.10.002.

Bertolazi, Alessandra Naimaier, Simone Chaves Fagondes, Leonardo Santos Hoff, Eduardo Giacomolli Dartora, Ilsis Cristine da Silva Miozzo, Maria Emília Ferreira de Barba, e Sérgio Saldanha Menna Barreto. 2011. "Validation of the Brazilian Portuguese Version of the Pittsburgh Sleep Quality Index". *Sleep Medicine* 12 (1): 70–75. https://doi.org/10.1016/j.sleep.2010.04.020.

Bloemer, Jenna, Priyanka D. Pinky, Manoj Govindarajulu, Hao Hong, Robert Judd, Rajesh H. Amin, Timothy Moore, Muralikrishnan Dhanasekaran, Miranda N. Reed, e Vishnu Suppiramaniam. 2018. "Role of Adiponectin in Central Nervous System Disorders". *Neural Plasticity* 2018: 4593530. https://doi.org/10.1155/2018/4593530.

Bolfarine, Heleno, e Wilton de Oliveira Bussab. 2005. *Elementos de amostragem*.

Boyce, Philip, e Anthea Hickey. 2005. "Psychosocial Risk Factors to Major Depression after Childbirth". *Social Psychiatry and Psychiatric Epidemiology* 40 (8): 605–12. https://doi.org/10.1007/s00127-005-0931-0.

Bravi, Francesca, Frank Wiens, Adriano Decarli, Alessia Dal Pont, Carlo Agostoni, e Monica Ferraroni. 2016. "Impact of Maternal Nutrition on Breast-Milk Composition: A Systematic Review". *The American Journal of Clinical Nutrition* 104 (3): 646–62. https://doi.org/10.3945/ajcn.115.120881.

Buist, A., D. Westley, e C. Hill. 1999. "Antenatal Prevention of Postnatal Depression". *Archives of Women's Mental Health* 1 (4): 167–73. https://doi.org/10.1007/s007370050024.

Camargos, Ana Cristina R., Vanessa A. Mendonça, Katherine S. C. Oliveira, Camila Alves de Andrade, Hércules Ribeiro Leite, Sueli Ferreira da Fonseca, Erica Leandro Marciano Vieira, Antônio Lúcio Teixeira Júnior, e Ana Cristina Rodrigues Lacerda. 2017. "Association between Obesity-Related Biomarkers and Cognitive and Motor Development in Infants". *Behavioural Brain Research* 325 (Pt A): 12–16. https://doi.org/10.1016/j.bbr.2017.02.030.

Çatlı, Gönül, Nihal Olgaç Dündar, e Bumin Nuri Dündar. 2014. "Adipokines in Breast Milk: An Update". *Journal of Clinical Research in Pediatric Endocrinology* 6 (4): 192–201. https://doi.org/10.4274/Jcrpe.1531.

Codoñer-Franch, Pilar, María T. Hernández-Aguilar, Almudena Navarro-Ruiz, Ana B. López-Jaén, Cintia Borja-Herrero, e Victoria Valls-Bellés. 2013. "Diet

Supplementation during Early Lactation with Non-Alcoholic Beer Increases the Antioxidant Properties of Breastmilk and Decreases the Oxidative Damage in Breastfeeding Mothers". *Breastfeeding Medicine: The Official Journal of the Academy of Breastfeeding Medicine* 8 (abril): 164–69. https://doi.org/10.1089/bfm.2012.0059.

Costa, Caroline dos Santos, Franciane Rocha de Faria, Kamila Tiemann Gabe, Isabela Fleury Sattamini, Neha Khandpur, Fernanda Helena Marrocos Leite, Eurídice Martínez Steele, Maria Laura da Costa Louzada, Renata Bertazzi Levy, e Carlos Augusto Monteiro. 2021. "Escore Nova de consumo de alimentos ultraprocessados: descrição e avaliação de desempenho no Brasil". *Revista de Saúde Pública* 55 (abril). https://doi.org/10.11606/s1518-8787.2021055003588.

Cox, J L, J M Holden, e R Sagovsky. 1987. "Detection of Postnatal Depression. Development of the 10-Item Edinburgh Postnatal Depression Scale". *The British Journal of Psychiatry: The Journal of Mental Science* 150 (junho): 782–86.

Fields, David A., Paul B. Higgins, e Gary R. Hunter. 2004. "Assessment of Body Composition by Air-Displacement Plethysmography: Influence of Body Temperature and Moisture". *Dynamic Medicine: DM* 3 (1): 3. https://doi.org/10.1186/1476-5918-3-3.

Filgueiras, Alberto, Pedro Pires, Silvia Maissonette, e J. Landeira-Fernandez. 2013. "Psychometric Properties of the Brazilian-Adapted Version of the Ages and Stages Questionnaire in Public Child Daycare Centers". *Early Human Development* 89 (8): 561–76. https://doi.org/10.1016/j.earlhumdev.2013.02.005.

Garza, Jacob C., Ming Guo, Wei Zhang, e Xin-Yun Lu. 2008. "Leptin Increases Adult Hippocampal Neurogenesis in Vivo and in Vitro". *The Journal of Biological Chemistry* 283 (26): 18238–47. https://doi.org/10.1074/jbc.M800053200.

Gomes-Oliveira, Marcio Henrique, Clarice Gorenstein, Francisco Lotufo Neto, Laura Helena Andrade, e Yuan Pang Wang. 2012. "Validation of the Brazilian Portuguese Version of the Beck Depression Inventory-II in a Community Sample". *Brazilian Journal of Psychiatry* 34 (dezembro): 389–94. https://doi.org/10.1016/j.rbp.2012.03.005.

Gorska-Ciebiada, Malgorzata, Malgorzata Saryusz-Wolska, Anna Borkowska, Maciej Ciebiada, e Jerzy Loba. 2016. "Adiponectin, leptin and IL-1 β in elderly diabetic patients with mild cognitive impairment". *Metabolic Brain Disease* 31: 257–66. https://doi.org/10.1007/s11011-015-9739-0.

Grantham-McGregor, Sally, Yin Bun Cheung, Santiago Cueto, Paul Glewwe, Linda Richter, e Barbara Strupp. 2007. "Developmental Potential in the First 5 Years for Children in Developing Countries". *The Lancet* 369 (9555): 60–70. https://doi.org/10.1016/S0140-6736(07)60032-4.

Gross, Rachel S, Nerissa K Velazco, Rahil D Briggs, e Andrew D Racine. 2013. "Maternal Depressive Symptoms and Child Obesity in Low-Income Urban Families". *Academic Pediatrics* 13 (4): 356–63. https://doi.org/10.1016/j.acap.2013.04.002.

Gunstad, John, Mary Beth Spitznagel, Therese A. Keary, Ellen Glickman, Thomas Alexander, Jessica Karrer, Kelly Stanek, Lynn Reese, e Judi Juvancic-Heltzel. 2008. "Serum Leptin Levels Are Associated with Cognitive Function in Older Adults". *Brain Research* 1230 (setembro): 233–36. https://doi.org/10.1016/j.brainres.2008.07.045.

Guo, M., C. Li, Y. Lei, S. Xu, D. Zhao, e X.-Y. Lu. 2017. "Role of the Adipose PPARγ-Adiponectin Axis in Susceptibility to Stress and Depression/Anxiety-Related

Behaviors". *Molecular Psychiatry* 22 (7): 1056–68. https://doi.org/10.1038/mp.2016.225.

Harvey, Jenni. 2007. "Leptin regulation of neuronal excitability and cognitive function". *Current Opinion in Pharmacology* 7 (6–3): 643–47. https://doi.org/10.1016/j.coph.2007.10.006.

Hibbeln, Joseph R. 2002. "Seafood consumption, the DHA content of mothers' milk and prevalence rates of postpartum depression: a cross-national, ecological analysis". *Journal of affective disorders* 69 (1): 15–30.

Holden, Karen F., Karla Lindquist, Frances A. Tylavsky, Caterina Rosano, Tamara B. Harris, e Kristine Yaffe. 2009. "Serum leptin level and cognition in the elderly: Findings from the Health ABC Study". *Neurobiology of aging* 30 (9): 1483–89. https://doi.org/10.1016/j.neurobiolaging.2007.11.024.

Jensen, Sarah K G, Iroise Dumontheil, e Edward D Barker. 2013. "Developmental Inter-Relations between Early Maternal Depression, Contextual Risks, and Interpersonal Stress, and Their Effect on Later Child Cognitive Functioning". *Depression and Anxiety*, setembro. https://doi.org/10.1002/da.22147.

Lanza di Scalea, Teresa, e Katherine L Wisner. 2009. "Antidepressant Medication Use during Breastfeeding". *Clinical Obstetrics and Gynecology* 52 (3): 483–97. https://doi.org/10.1097/GRF.0b013e3181b52bd6.

Lee, Thomas Ho-yin, Kenneth King-yip Cheng, Ruby Lai-chong Hoo, Parco Ming-fai Siu, e Suk-yu Yau. 2019. "The Novel Perspectives of Adipokines on Brain Health". *International Journal of Molecular Sciences* 20 (22): 5638. https://doi.org/10.3390/ijms20225638.

Li, Nan, Tye E. Arbuckle, Gina Muckle, Bruce P. Lanphear, Michel Boivin, Aimin Chen, Linda Dodds, et al. 2019. "Associations of Cord Blood Leptin and Adiponectin with Children's Cognitive Abilities". *Psychoneuroendocrinology* 99: 257–64. https://doi.org/10.1016/j.psyneuen.2018.10.021.

Liu, J., M. Guo, D. Zhang, S.-Y. Cheng, M. Liu, J. Ding, P. E. Scherer, F. Liu, e X.-Y. Lu. 2012. "Adiponectin is critical in determining susceptibility to depressive behaviors and has antidepressant-like activity". *Proceedings of the National Academy of Sciences* 109 (30): 12248–53. https://doi.org/10.1073/pnas.1202835109.

Mello, Marco Túlio de, Ana R. Dâmaso, Hanna Karen M. Antunes, Kãli O. Siqueira, Marise Lazaretti Castro, Sheila V. Bertolino, Sérgio G. Stella, e Sérgio Tufik. 2005. "Avaliação da composição corporal em adolescentes obesos: o uso de dois diferentes métodos". *Revista Brasileira de Medicina do Esporte* 11 (5): 267–70. https://doi.org/10.1590/S1517-86922005000500004.

Oates, Margaret. 2003. "Suicide: The Leading Cause of Maternal Death". *The British Journal of Psychiatry* 183 (4): 279–81. https://doi.org/10.1192/bjp.183.4.279.

O'Malley, Dervla, Neil MacDonald, Sarah Mizielinska, Christopher N. Connolly, Andrew J. Irving, e Jenni Harvey. 2007. "Leptin promotes rapid dynamic changes in hippocampal dendritic morphology". *Molecular and Cellular Neurosciences* 35 (4): 559–72. https://doi.org/10.1016/j.mcn.2007.05.001.

Otto, S.J, R.H.M de Groot, e G Hornstra. 2003. "Increased risk of postpartum depressive symptoms is associated with slower normalization after pregnancy of the functional docosahexaenoic acid status". *Prostaglandins, Leukotrienes and Essential Fatty Acids* 69 (4): 237–43. https://doi.org/10.1016/S0952-3278(03)00090-5.

Rebelo, Fernanda, Dayana R Farias, Claudio J Struchiner, e Gilberto Kac. 2016. "Plasma Adiponectin and Depressive Symptoms during Pregnancy and the

Postpartum Period: A Prospective Cohort Study". *Journal of Affective Disorders* 194 (abril): 171–79. https://doi.org/10.1016/j.jad.2016.01.012.

Rebelo, Fernanda, Thatiana de Jesus Pereira Pinto, Ana Beatriz Franco-Sena, Jaqueline Lepsch, Camila Benaim, Claudio José Struchiner, e Gilberto Kac. 2015. "Plasma Adiponectin Is Inversely Associated with Antenatal Anxiety: Results from a Brazilian Cohort". *Psychoneuroendocrinology* 51 (janeiro): 92–100. https://doi.org/10.1016/j.psyneuen.2014.09.015.

Santos, Iná S., Alicia Matijasevich, Beatriz Franck Tavares, Aluísio J. D. Barros, Iara Picinini Botelho, Catherine Lapolli, Pedro Vieira da Silva Magalhães, Ana Paula Pereira Neto Barbosa, e Fernando C. Barros. 2007. "Validation of the Edinburgh Postnatal Depression Scale (EPDS) in a Sample of Mothers from the 2004 Pelotas Birth Cohort Study". *Cadernos De Saúde Pública* 23 (11): 2577–88.

Savino, Francesco, e Stefania A. Liguori. 2008. "Update on Breast Milk Hormones: Leptin, Ghrelin and Adiponectin". *Clinical Nutrition (Edinburgh, Scotland)* 27 (1): 42–47. https://doi.org/10.1016/j.clnu.2007.06.006.

Sit, Dorothy, James M. Perel, Stephen R. Wisniewski, Joseph C. Helsel, James F. Luther, e Katherine L. Wisner. 2011. "Mother-Infant Antidepressant Concentrations, Maternal Depression, and Perinatal Events". *The Journal of Clinical Psychiatry* 72 (7): 994–1001. https://doi.org/10.4088/JCP.10m06461.

Textor, Johannes, Juliane Hardt, e Sven Knüppel. 2011. "DAGitty: A Graphical Tool for Analyzing Causal Diagrams". *Epidemiology (Cambridge, Mass.)* 22 (5): 745. https://doi.org/10.1097/EDE.0b013e318225c2be.

Venancio, Sonia I., Maria M. L. Escuder, Sílvia R. D. M. Saldiva, e Elsa R. J. Giugliani. 2010. "Breastfeeding practice in the Brazilian capital cities and the Federal District: current status and advances". *Jornal de Pediatria* 86 (4): 317–24. https://doi.org/10.1590/S0021-75572010000400012.

Villar, José, Leila Cheikh Ismail, Cesar G. Victora, Eric O. Ohuma, Enrico Bertino, Doug G. Altman, Ann Lambert, et al. 2014. "International Standards for Newborn Weight, Length, and Head Circumference by Gestational Age and Sex: The Newborn Cross-Sectional Study of the INTERGROWTH-21st Project". *Lancet (London, England)* 384 (9946): 857–68. https://doi.org/10.1016/S0140-6736(14)60932-6.

Yildiz, Gazi, Mehmet Baki Senturk, Pinar Yildiz, Yusuf Cakmak, Mehmet Sukru Budak, e Erbil Cakar. 2017. "Serum Serotonin, Leptin, and Adiponectin Changes in Women with Postpartum Depression: Controlled Study". *Archives of Gynecology and Obstetrics* 295 (4): 853–58. https://doi.org/10.1007/s00404-017-4313-0.

Young, B. E., C. Levek, R. M. Reynolds, M. C. Rudolph, P. MacLean, T. L. Hernandez, J. E. Friedman, e N. F. Krebs. 2018. "Bioactive Components in Human Milk Are Differentially Associated with Rates of Lean and Fat Mass Deposition in Infants of Mothers with Normal vs. Elevated BMI". *Pediatric Obesity* 13 (10): 598–606. https://doi.org/10.1111/ijpo.12394.

Zhang, D., M. Guo, W. Zhang, e X.-Y. Lu. 2011. "Adiponectin Stimulates Proliferation of Adult Hippocampal Neural Stem/Progenitor Cells through Activation of P38 Mitogen-Activated Protein Kinase (P38MAPK)/Glycogen Synthase Kinase 3 (GSK-3 )/ -Catenin Signaling Cascade". *Journal of Biological Chemistry* 286 (52): 44913–20. https://doi.org/10.1074/jbc.M111.310052.

**Anexo 1 – Escala de Depressão Pós-parto de Edinburgh (EPDS)**

| Questionário Edinburgh | | |
|---|---|---|
| **Você teve um bebê e nós gostaríamos de saber como você está se sentindo. Para isso, peço que você responda a opção que mais se aproxima de como você se sentiu NOS ÚLTIMOS SETE DIAS, e não apenas como você se sente hoje.** | | |
| **1** | **Eu tenho sido capaz de rir e achar graça das coisas.** | 0 [ ] **Como eu sempre fiz.**<br>1 [ ] **Não tanto quanto antes.**<br>2 [ ] **Sem dúvida, menos que antes.**<br>3 [ ] **De jeito nenhum.** |
| **2** | **Eu tenho pensado no futuro com alegria.** | 0 [ ] **Sim, como de costume.**<br>1 [ ] **Um pouco menos que de costume.**<br>2 [ ] **Muito menos que de costume.**<br>3 [ ] **Praticamente não.** |
| **3** | **Eu tenho me culpado sem razão quando as coisas dão errado.** | 0 [ ] **Não, de jeito nenhum.**<br>1 [ ] **Raramente.**<br>2 [ ] **Sim, às vezes.**<br>3 [ ] **Sim, muito frequentemente.** |
| **4** | **Eu tenho ficado ansiosa ou preocupada sem uma boa razão.** | 3 [ ] **Sim, muito seguido.**<br>2 [ ] **Sim, às vezes.**<br>1 [ ] **De vez em quando.**<br>0 [ ] **Não, de jeito nenhum.** |
| **5** | **Eu tenho me sentido assustada ou em pânico sem um bom motivo.** | 3 [ ] **Sim, muito seguido.**<br>2 [ ] **Sim, às vezes.**<br>1 [ ] **Raramente.**<br>0 [ ] **Não, de jeito nenhum.** |
| **6** | **Eu tenho me sentido sobrecarregada pelas tarefas e acontecimentos do meu dia-a-dia** | 3 [ ] **Sim. Na maioria das vezes eu não consigo lidar bem com eles.**<br>2 [ ] **Sim. Algumas vezes não consigo lidar bem como antes.**<br>1 [ ] **Não. Na maioria das vezes consigo lidar bem com eles.**<br>0 [ ] **Não. Eu consigo lidar com eles tão bem quanto antes.** |
| **7** | **Eu tenho me sentido tão infeliz que eu tenho tido dificuldade de dormir.** | 3 [ ] **Sim, na maioria das vezes.**<br>2 [ ] **Sim, algumas vezes.**<br>1 [ ] **Raramente.**<br>0 [ ] **Não, nenhuma vez.** |
| **8** | **Eu tenho me sentido triste ou muito mal.** | 3 [ ] **Sim, na maioria das vezes.**<br>2 [ ] **Sim, muitas vezes.**<br>1 [ ] **Raramente.**<br>0 [ ] **Não, de jeito nenhum.** |
| **9** | **Eu tenho me sentido tão triste que tenho chorado.** | 3 [ ] **Sim, a maior parte do tempo.**<br>2 [ ] **Sim, muitas vezes.**<br>1 [ ] **Só de vez em quando.**<br>0 [ ] **Não, nunca.** |
| **10** | **Eu tenho pensado em fazer alguma coisa contra mim mesma.** | 3 [ ] **Sim, muitas vezes.**<br>2 [ ] **Às vezes.**<br>1 [ ] **Raramente.**<br>0 [ ] **Nunca.** |

**Anexo 2 – Folder de divulgação**

**Já imaginou poder acompanhar a sua composição corporal e o desenvolvimento cognitivo e motor do seu bebê durante o próximo ano com as melhores avaliações disponíveis?**

Se você tem de 20 a 45 anos de idade e teve parto a termo (37 semanas ou mais), converse com uma das pesquisadoras do Projeto APPLE na sua visita ao banco de leite.

**Traga uma roupa justa (top e short) para avaliação da sua composição corporal.**
