# Supplementary material for: Plasma and breast milk adipokines in women across the first year postpartum and their association with maternal depressive symptoms and infant neurodevelopment: Protocol for the APPLE prospective cohort study
Source: PLoS One. 2024 Oct 25;19(10):e0310847. doi: 10.1371/journal.pone.0310847 (PMC11508165; doi:10.1371/journal.pone.0310847)
Supplement: S2 Protocol — (PDF) [file pone.0310847.s003.pdf]

Machine Translated by Google

RESEARCH PROJECT

Title: Adipocytokines in plasma and milk of nursing mothers in the first year postpartum: association with depressive symptoms and infant cognitive and motor development

Project coordination: Fernanda Rebelo dos Santos

EXECUTING INSTITUTION: FOUNDATION OSWALDO CRUZ / NATIONAL INSTITUTE OF WOMEN'S, CHILDREN'S, AND HEALTH HEALTH ADOLESCENT FERNANDES FIGUEIRA.

Machine Translated by Google

Summary

Introduction: The role of adipocytokines in the central nervous system has been the focus of several studies in the last decade. In vitro studies demonstrate the role of adiponectin and leptin in stimulating neurogenesis, which is a possible mechanism by which These adipocytokines can act both in stimulating cognitive development and infant motor skills, as well as in the inhibition of depressive symptoms in postpartum women. **Goals:** Evaluate the correlation between adipocytokines (adiponectin and leptin) in plasma and milk mothers and their association with: child cognitive and motor development at 6 and 12 months of age; and the mental health of the postpartum woman throughout the first year postpartum. **Methods:** This is a prospective cohort with four waves of follow-up. To the postpartum women and their children, newborns in the IFF maternity ward, will be captured during the visit follow-up to the Human Milk Bank, up to 15 days postpartum. O Follow-up will take place at 2, 6 and 12 months postpartum. Visits will include collection of maternal blood and milk, application of the Edinburgh scale to assess mental health maternal, application of the Bayley-III child development scale, anthropometry maternal and child health and assessment of reproductive history, breastfeeding and data socioeconomic and demographic. **Expected results:** The proposal aims to, firstly, to provide support for clarifying the role of adipocytokines in nervous system of children and postpartum women and, consequently, promote improvement in the care and quality of life of these groups. Additionally, the project will provide data on type of birth, child physical growth and variation in maternal body composition in first year postpartum, which are of great relevance to public health and, when inter-related, may generate important results and contribute to the existing literature. Furthermore, the project aims to disseminate results at national events and international publications and the publication of scientific articles, contributing to increasing qualitatively and quantitatively the national scientific production, in addition to the incorporation of scientific initiation, master's and doctorate students, contributing to the formation of highly qualified human resources.

Machine Translated by Google

1. Introduction

Human milk contains several nutrients, cytokines, peptides, enzymes, cells, immunoglobulins, proteins and steroids capable of meeting the infant's needs (Ballard and Morrow 2013). Some of these compounds are synthesized by the glands mammary glands while others are extracted from maternal plasma. The nutritional status maternal health, the use of supplements and food consumption are some of the factors that can modify plasma concentrations of biomarkers and, consequently, the milk composition (Codoñer-Franch et al. 2013; Bravi et al. 2016; Young et al. 2018).

Among the biomarkers already identified in breast milk are adiponectin and leptin, also known as obesity-related biomarkers or adipocytokines, as they are secreted by adipose cells (Çatlÿ, Olgaç Dündar, and Dündar 2014). It is believed that these cytokines present in breast milk have hormonal activities in various tissues of the neonate while its endocrine system itself is not completely functional, performing functions in metabolism energy and regulation of body composition (Savino and Liguori 2008).

Additionally, studies suggest that adipocytokines may play activities in the brain, such as stimulation of neuronal genesis and excitability of the hippocampus, participating in activities such as learning and memory (Harvey 2007; Liu et al. 2012; O'Malley et al. 2007; Bloemer et al. 2018; Lee et al. 2019). A few years ago the association of adipocytokines with cognitive function has already been studied in other population groups, such as adults and the elderly (Gorska-Ciebiada et al. 2016; Gunstad et al. 2008; Holden et al. 2009). More recently, some studies have presented data on child population.

In a cross-sectional study with 50 participants carried out in Brazil, concentrations plasma leptin levels were inversely associated with the development cognitive in children between 6 and 24 months of age (Camargos et al. 2017). Using data from two cohorts of pregnant women in the United States and Canada, Li et al. (2019) found an association between adiponectin in umbilical cord blood and intelligence and memory coefficient in children aged 3, 5 and 8 years. Nonetheless, To date, no studies have been found investigating the association of non-maternal adipocytokines and the cognitive development of infants during first year of life.

Machine Translated by Google

When it comes to maternal outcomes associated with concentrations of adipocytokines in postpartum women, more clarification is needed regarding health mental. The literature presents some evidence that individuals with conditions such as depression and anxiety can have altered plasma concentrations of adipocytokines (Yildiz et al. 2017; Rebelo et al. 2015; Guo et al. 2017). However, data on the relationship between adipocytokines and mental health in the postpartum period are scarce and inconclusive (Rebelo et al. 2016).

Thus, the present project seeks to clarify the relationship between adipocytokines in maternal plasma and milk, postpartum mental health and cognitive and motor development of the infant, based on the hypothesis that plasma concentrations of adiponectin and leptin are associated with the mental health status of the nursing mother and correlated with concentrations of the same adipocytokines in breast milk. In turn, ingestion of milk with higher concentrations of adiponectin and lower concentrations of leptin induces greater neuronal stimulation of the infant, providing better cognitive and motor.

2. Justification

This proposal aims to produce high-quality scientific knowledge quality from carrying out a translational study, with a prospective design throughout the first year postpartum. It is interesting to highlight that the project focuses on a topic of high scientific relevance and with a health impact important. Both the exposures and outcomes being studied are of great importance for the area of maternal and child health and were little studied until the moment.

The role of adipocytokines in the central nervous system has been the focus of several studies in the last decade. In vitro studies demonstrate the role of adiponectin and leptin in stimulating neurogenesis by regulating the proliferation of hippocampal cells in a time- and dose-dependent manner (Zhang et al. 2011; Garza et al. 2008). Therefore, the stimulation of neurogenesis is a possible mechanism by which these adipocytokines can act both in stimulating the infant's cognitive and motor development, as well as in inhibition of depressive symptoms in postpartum women.

Machine Translated by Google

The period in which brain growth reaches greater speed and presents Greater plasticity occurs in the last trimester of pregnancy and in the first two years of pregnancy. life. Thus, the first thousand days of life, from conception to 2 years of age, are identified as crucial in the development trajectory, being determinants of the state future health, and are associated with greater cognitive and learning capacity during adulthood (Grantham-McGregor et al. 2007). In this way, the identification of factors that may be associated with neurodevelopment in the first year of life, considered a window of opportunities for interventions, it is of great relevance for contribute to the health and quality of life of the population.

Postpartum depression is especially problematic because it affects not only the women's health, as well as negatively interfering with children's health and having drug treatment is made difficult, as there is transmission of the drug to the child, through breast milk (Gross et al. 2013; Jensen, Dumontheil, and Barker 2013; Lanza di Scalea and Wisner 2009; Sit et al. 2011). Mothers with depression often have the compromised physical health and high-risk behaviors such as alcohol abuse and other substances. These women are less likely to take adequate care of their their own needs and those of their children and are more resistant to seeking and receiving postnatal care or adhering to medical prescriptions (Alder et al. 2007). Furthermore, the suicide, whose main risk factor is depression, is the main cause of maternal death in developed countries (Oates 2003).

Given the scarcity of studies on this subject, this proposal stands out for its its innovative potential and ability to generate evidence capable of assisting in understanding and preventing developmental problems in childhood and throughout of the life cycle, in addition to assisting in the search for new strategies to prevent and treat baby blues.

3. Objectives

3.1 General objective

To evaluate the correlation between adipocytokines (adiponectin and leptin) in plasma and breast milk and its association with: child cognitive and motor development at 6 and 12 months of age; and the mental health of the postpartum woman throughout the first year postpartum.

3.2 Specific objectives

- Describe and correlate adipocytokine concentrations in plasma and milk mothers in the first year postpartum, according to selected variables, including maternal weight variation and type of delivery;
- Describe *children's* cognitive development and physical growth in the first year of life according to selected maternal variables;
- Describe the occurrence of depressive symptoms in postpartum women throughout the first year postpartum according to selected maternal variables;
- Evaluate the association between the mental health of breastfeeding women and concentrations of maternal adiponectin and leptin;
- Evaluate the association between mental health of postpartum women and physical and cognitive and motor development of infants in the first year of life;
- Evaluate the association between maternal adipocytokines and physical growth and cognitive and motor development of infants in the first year of life;
- Assess the mediating role of adipocytokines in breast milk and mental health of postpartum women in the relationship between adipocytokines in maternal plasma and growth physical and cognitive and motor development of the infant in the first year of life.
- Evaluate the association between sleep quality and mother-baby bonding with health maternal mental;
- Evaluate the association between the consumption of ultra-processed foods and the concentration of adipocytokines in plasma and milk of lactating women;
- Evaluate the validity of monitoring the development milestones of the Child Health Booklet to identify developmental deficits childish.
- Describe and correlate concentrations of inflammatory biomarkers and vitamin D metabolites in maternal plasma and milk in the first year postpartum,

Machine Translated by Google

according to selected variables, including maternal weight variation, type of birth and consumption of ultra-processed foods;

- Evaluate the association between concentrations of inflammatory biomarkers and vitamin D metabolites in maternal milk and blood and depressive symptoms in postpartum women;
- Evaluate the association between concentrations of inflammatory biomarkers and vitamin D metabolites in maternal milk and blood and the development cognitive and motor at 6 and 12 months;

4. Goals

i. Provide support for clarifying the role of adipocytokines in the system nervous system of children and postpartum women and, consequently, promote improvement in care and quality of life for these groups;

ii. Provide data on type of birth, child physical growth and weight variation and maternal body composition in the first year postpartum. These data, from great relevance for public health, when interrelated, they can generate important results and contribute to the existing literature.

iii. Disseminate the results obtained in local seminars, national congresses and international and through the publication of scientific articles, contributing to qualitatively and quantitatively increase national scientific production;

iv. Enhance the training of human resources for the area of research and teaching, through dialogue, on a multidisciplinary basis, between the team of researchers and doctoral, master's and scientific initiation students who will be incorporated into team;

v. Stimulate interaction between departments at Instituto Fernandes Figueira (IFF) involved in this proposal, promoting the exchange of knowledge, encouraging the development of future projects on a multidisciplinary and improving the assistance provided to the Institution's users.

5. Methods

To facilitate communication, the research title "Adipocytokines in plasma and milk _ nursing mothers in the first year postpartum: association with depressive symptoms and cognitive and motor development of the *infant"* will be abbreviated with the acronym APPLE.

This is a prospective cohort with four waves of follow-up: immediate postpartum (up to 15 days postpartum), 2nd month postpartum, 6th month postpartum and 12th month postpartum. will be adult breastfeeding women and their children who are in the period up to 15 days postpartum. Participants will be recruited on two fronts: (1) dissemination in the IFF rooming-in, with subsequent active search for postpartum women eligible individuals who return for childcare monitoring at the Milk Bank Human (BLH) from IFF; (2) outreach to personal contacts of the project team in snowball tactic, where interested parties will get in touch by email or phone to assessment of eligibility and scheduling of the first assessment.

Monitoring will take place at the HMB (milk collection), at the pediatrics service (evaluation child development) and in the Nutrition and Metabolism Laboratory (assessment of body composition, blood collection and application of questionnaires). The detailing of the variables obtained in each follow-up wave of the study can be observed in the **Figure** 1. Recruitment will follow the eligibility criteria described in **Table** 1.

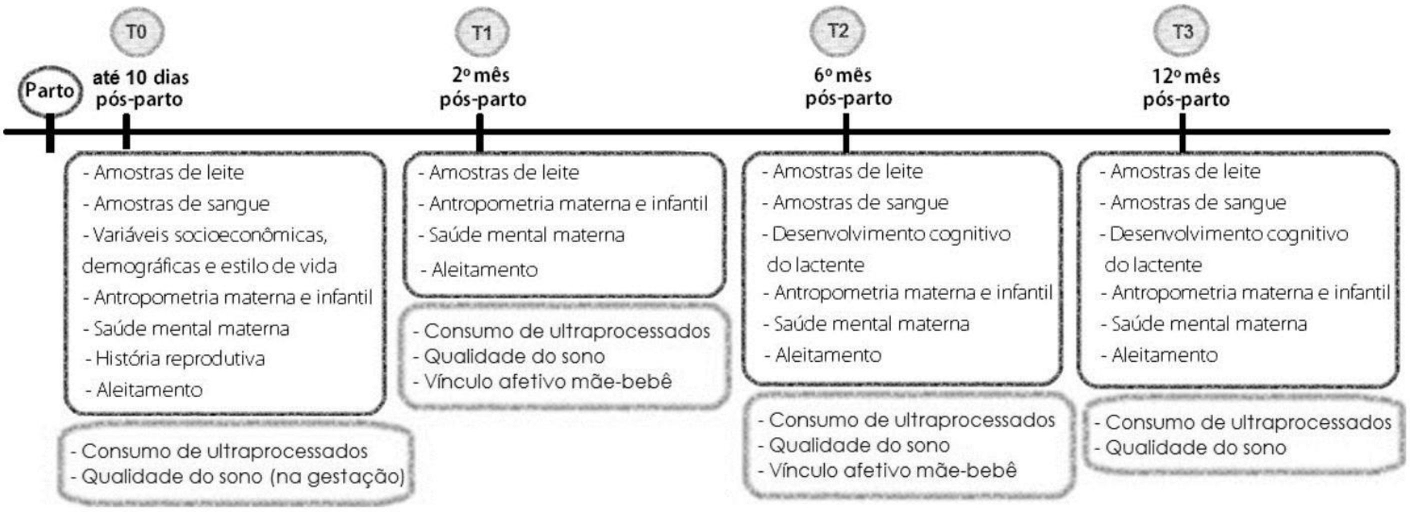

Figure 1. Data obtained in each wave of cohort follow-up.

Table 1. Eligibility criteria.

**Inclusion criteria ÿ Will be selected breastfeeding women who meet the following eligibility criteria at the time of recruitment:**	**Exclusion criteria ÿ Must be excluded to the breastfeeding criteria:**
1. Being up to 15 days postpartum;	1. Whose newborn had a premature birth;
two. Be between 20 and 45 years of age;	2. Whose newborn has any pathology or weight not appropriate for gestational age (<p5 or >p95, Villar et al. 2014);
3. Being free from chronic diseases communicable diseases such as high blood pressure and diabetes, except obesity;	3. Using antidepressant or other medication psychiatric medication.
4. Be free from infectious diseases, such as HIV;	4. Who had a twin pregnancy;
5. Be exclusively breastfeeding, defined such as the exclusive supply of breast milk, without teas, or water, in the 5 days preceding entry into the study.	5. Whose newborn is diagnosed with any aggravation or malformation that is known to cause motor and/or cognitive deficits;
6. Reside in the city of Rio de Janeiro;	6. Who had positive serology during pregnancy for Syphilis, Rubella, Toxoplasmosis, CMV or Zika;
7. Newborn with Apgar ÿ7 in the fifth minute;	7. Whose newborn was hospitalized in neonatal ICU;
8. Proficiency in the Portuguese language.	9. Women who had a pregnancy with a uterus replacement/temporary transfer of the uterus *("solidarity belly")* or who will give the baby up for adoption.

5.1 Study variables

Machine Translated by Google

• Blood and breast milk samples

Human milk (minimum 10 ml and maximum 20 ml) will be extracted manually, one sample being collected at the beginning and another at the end of breastfeeding, by the breastfeeding woman. These samples will be stored and transported, under refrigeration, within a maximum of 2 hours to the place where the analyzes were carried out and homogenized in a ultrasonic (20 Hz for 1.5 seconds/ml). Afterwards, they will be separated into aliquots and stored at -20ºC until processing and analysis. Samples will be obtained at all follow-up visits or until the postpartum woman no longer has any milk.

Blood samples will be extracted at visits T0, T2 and T3 in a collection tube containing EDTA for determining the concentration of adipocytokines using kits ELISA commercials. The samples will be centrifuged (5,000 rpm/5 minutes) and the plasma will be separated into cryotubes and stored in a freezer at -20°C until the date of carrying out the measurements. analyses.

In addition to adipocytokines, inflammatory biomarkers and vitamin D metabolites. Inflammatory biomarkers such as oxylipins and fatty acids, will be analyzed by high-resolution liquid chromatography coupled to mass spectrometry (HPLC-MS/MS)(Durand et al. 2001; 2002). The metabolites of Vitamin D will be analyzed by HPLC-MS/MS (Agilent® 1260 Infinity), with a Lux® cellulose F5 column (Phenomenex), coupled mass spectrometer – AB Sciex QTrap® 5500, operating in positive mode (Satoh et al. 2016).

• Cognitive and motor development of the infant

Cognitive and motor development will be assessed using the Bayley scales of Child Development (BSID-III) in the 6th and 12th month after birth (margin of 15 days more or less from the exact date). This instrument is intended for evaluating of children aged 1-42 months and composed of the following scales: (i) Cognitive Scale, which evaluates sensorimotor development, exploration and manipulation, formation of concepts and memory; (ii) Language Scale, composed of communication items receptive and expressive, forming two distinct sub-tests; (iii) Motor Scale, which evaluates general motor skills and fine motor skills; (iv) Socio-emotional scale, which identifies the main milestones of social and emotional development in certain ages; (v) Adaptive Behavior Scale, which accesses functional abilities of the child's daily life and considers communication, home life, health, safety and leisure.

Machine Translated by Google

Additionally, the development milestones present in the Child Health Booklet. This assessment is performed routinely in the clinic pediatric and verify its validity based on comparison with the results of the BSID-III (gold standard) will be another important piece of data generated by this study.

The result of the assessment will be communicated to the responsible pediatrician, so that appropriate measures are taken, allowing referral to a specialist if necessary.

• Maternal mental health

Maternal mental health will be monitored at all follow-up waves: up to 15 days, 2, 6 and 12 months postpartum. During the first consultation, data will be collected about the history of depression. A validated version of the Postgraduate Depression Scale will be used. Edinburgh calving (EPDS). The scale was developed by Cox et al. (Cox, Holden, and Sagovsky 1987) and translated and validated into Portuguese by Santos et al. (Santos et al. 2007) and consists of a self-recording instrument composed of 10 statements, with options scored from 0 to 3 according to the presence and intensity of the depressive symptom (Annex 1).

There are several studies that cite and use the EPDS as it is a questionnaire easy application and understanding for women, in addition to being quite efficient in identification of postpartum depression (Boyce and Hickey 2005; Buist, Westley, and Hill 1999; Hibbeln 2002; Otto, de Groot, and Hornstra 2003). The participant will be encouraged to complete the questionnaire questions alone, but you will have the help of a trained interviewer to clarify any doubts or assist you in reading the questions and filling in the answers if she is unable to execute it correctly. independent.

Additionally, there will be an assessment of depression using the Inventory of Beck Depression-II (BDI-II) at 6 and 12 months postpartum. This is because the validation of The Brazilian version of the EPDS was carried out only with women up to 3 months postpartum. The BDI-II is an instrument widely used around the world and validated for Brazilian population (Gomes-Oliveira et al. 2012). The application will be carried out by trained psychologist.

Machine Translated by Google

The result of the evaluation will be communicated to the responsible gynecologist, so that the necessary measures inherent to adequate treatment are taken, allowing referral to a specialist if necessary.

• Socioeconomic, demographic, lifestyle and reproductive history variables

A structured questionnaire will be applied to determine the characteristics maternal socioeconomic and demographic data, as well as lifestyle data and reproductive factors that appear in the nursing mother's history: age, marital status, number of children, occupation, level of education, place of residence, type of housing, smoking, alcohol consumption and sex of the newborn; number of births, date of last birth, type of birth (specifying the reason for surgical delivery, when applicable) and duration of pregnancy.

• Maternal and child anthropometry

At the first consultation, data will be collected on the pre-pregnancy weight of the woman. For this, preference will be given to weight measured up to the 13th week of pregnancy recorded in the pregnant woman's monitoring booklet. If this record is non-existent, the reported weight will be recorded. For the newborn, the following data contained in the Child Health Booklet: birth weight, length at birth and head circumference at birth.

To evaluate the body composition of postpartum women and babies weighing up to 8kg the air displacement plethysmography method will be used, which evaluates the volume and body density by measuring the volume of air displaced by the body inside a closed chamber. It is a relatively recent, fast, safe, comfortable method (Fields, Higgins, and Hunter 2004). This technique is based on Boyle's law which is relies on the inverse relationship between pressure and volume to determine the composition body. Once this volume is defined, it is possible to apply the principles of densitometry to determine body composition by calculating body density (Mello et al. 2005). The plethysmograph, also called the adult BOD POD or PEA POD for babies, it is coupled to a computer that determines variations in air volume and of pressure inside the empty and occupied chamber. This equipment makes adjustments to pulmonary variables necessary to estimate body volume (Guedes, 1998). O The equipment will also provide the weight of the woman and child at each interview.

Machine Translated by Google

Height will be assessed directly using a standard stadiometer. aluminum (SN4010, Sanny®) with an accuracy of one millimeter during the first wave of follow-up.

When the child weighs more than 8kg (maximum capacity of the PEA POD), the The child's weight will be measured using a digital pediatric scale with capacity maximum 20kg. It is estimated that the assessment of children's body composition will only be possible up to 6 months. The length of the child will be measured using an infantometer, with the child lying down, with their feet supported on the fixed part of the instrument. The moving part will be properly positioned over the child's head. The measurement will be carried out in duplicate, using the average of the measurements. The perimeter will also be measured cephalic/frontoccipital circumference of the child, using an extendable measuring tape, corresponding to the maximum head circumference.

- Breastfeeding

A breastfeeding questionnaire will be administered in all waves of follow-up. The frequency of breast milk consumption will be estimated using 24-hour recall. The instrument used will be adapted from the II Survey of Prevalence of Breastfeeding in Brazilian Capitals and the Federal District, Ministry of Health (Venancio et al. 2010).

- Ultra-processed food consumption

Food consumption of ultra-processed foods will be assessed in all waves of follow-up using the Nova Score (Costa et al. 2021). This is a questionnaire self-administered, lasting around three minutes, which proved to be good performance for estimating the participation of ultra-processed foods in the Brazilian diet. The questionnaire will be sent electronically (email or WhatsApp) one week before of face-to-face consultations (except for the first consultation). For women who don't respond to the electronic questionnaire, it will be administered during the in-person consultation.

- Sleep quality

Machine Translated by Google

Sleep quality will be assessed using the Sleep Quality Index. Pittsburgh translated and validated for the Brazilian population (PSQI-BR)(Bertolazi et al. 2011). In the first wave of follow-up (up to 15 days postpartum) the questions will refer to to the gestational period. In the other waves, the woman will be asked to respond with based on the current period. The questionnaire will be sent electronically (email or WhatsApp) one week before face-to-face consultations (except for the first consultation). For women who do not respond to the electronic questionnaire, it will be administered during the in-person consultation.

- Mother-baby emotional bond

The mother-baby bond will be assessed in the second and third waves of follow-up (T1 and T2), using the translated version of *Postpartum Bonding Questionnaire* (PBQ)(Baldisserotto et al. 2018). The questionnaire will be sent via electronic (email or WhatsApp) one week before face-to-face consultations (except for first consultation). For women who do not respond to the electronic questionnaire, this will be applied during the in-person consultation.

5.2 Assistance to participants

The occurrence of depressive symptoms in women is the critical outcome that expects to be more recurrent during project monitoring. The women who are classified as having depressive symptoms on the EPDS scale or diagnosed with depression by the BDI-II will be referred for care at the Health Coordination Mental IFF (COJ), if they are enrolled at IFF. Participants who do not have registration at the IFF will be sent to the outpatient clinic of the Institute of Psychiatry at UFRJ (IPUB).

In the case of other critical results, the project team will be responsible for that participants receive adequate care. It should be noted that the team is multidisciplinary team, composed of professionals from the areas of pediatrics, psychiatry, neonatology, psychology, nutrition and nursing.

5.3 Sample size

To evaluate the differences in adipocytokines in human milk according to the child development, we will use as a reference the global score of cognitive development in the communication domain. Previous study accepts that the deviation Standard score is 13.6. (Filgueiras et al. 2013). We will consider a significant difference of 3 points. We accept a risk of 0.05 and want a statistical power of 95% to detect differences if they exist.

Applying the sample size determination formula based on a population mean estimate (n=[Zÿ/2.ÿ/E]²) we will have N = 79 (Bolfarine and Bussab 2005). If we estimate a loss to follow-up of approximately 20%, the sample N there will be 95 pairs of mothers and children.

5.4 Data entry and statistical analysis

The information collected will be entered into the database through double typing in the RedCap software, in data entry masks specifically developed for the study.

Statistical analyzes will be performed using the STATA statistical package. First, an evaluation of the data will be carried out using dispersion analysis. Data normality will be investigated using the Kolmogorov-Smirnoff test and non-normal variables will be subjected to a logarithmic transformation for their subsequent analysis by parametric tests. The proportions of categorical variables will be compared using the chi-square test and differences between variable means continuous tests will be tested with T-Student. For the correlation between continuous variables it will be Pearson's correlation coefficient was used (for example, correlation between adipocytokines in plasma and milk).

The main variables (adipocytokine concentrations, development and depressive symptoms) will be worked on continuously. For To evaluate the association between them, a longitudinal mixed effects model will be used (LME, acronym for Linear Mixed Effects), adjusted for confounding factors. The LME is a model capable of capturing changes between and within individuals, accommodating time-dependent and independent covariates, take into account the correlation between repeated measures on the same individual and allows for unbalanced time intervals. O

Machine Translated by Google

Postpartum time will be included as a fixed and random effect variable. If the answer is described by a non-linear function, a quadratic term will be inserted into the model.

To determine the model adjustment variables, the acyclic graph will be used directed (DAG, from the English acronym *Direct Acyclic Graph)* built using the online software DAGitty (Textor, Hardt, and Knüppel 2011). The objective of the DAG is to improve our understanding of the association between exposure and outcome and all possible confounding factors. This approach allows you to identify the minimum adjustment sufficiently defined to estimate the direct effect of plasma concentrations of adiponectin and cognitive and motor development in infants. A first version of the DAG can be seen in **Figure** 2.

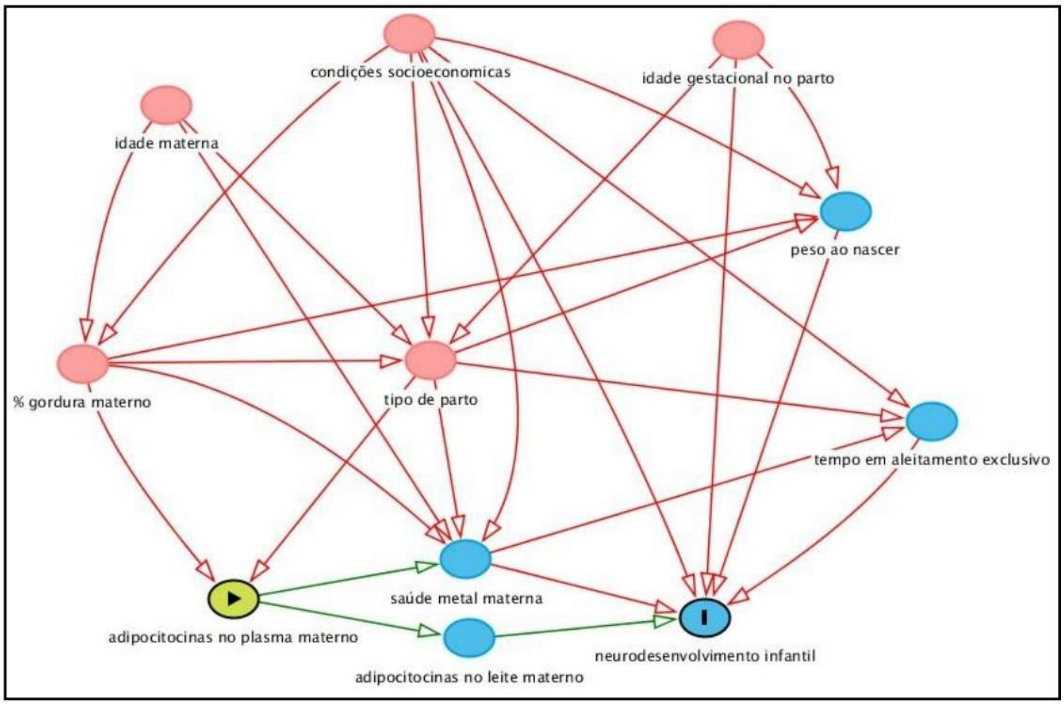

Figure 2. Directed acyclic graph of the relationship between adipocytokines in maternal plasma and child neurodevelopment. Minimum adjustment required: fat percentage mother and type of delivery.

Machine Translated by Google

5.5 Ethical issues

The project is in accordance with Resolutions nº 466, of December 12, 2012 and nº 510, of April 7, 2016. Participation in the research will be subject to signing two copies of the consent form, which will be obtained freely and spontaneously, after having made all the clarifications pertinent to this study.

6. Expected products, project impacts and feasibility of application of the knowledge produced.

The proposal aims, firstly, to promote advances in the understanding of relationships between plasma adipocytokines and breast milk, cognitive development and infant motor and maternal mental health. Additionally, the project will provide data on type of birth, child physical growth and maternal weight change in the first year post-childbirth. These data, of great relevance to public health, when interrelated, may generate important results and contribute to the existing literature.

Some in vitro studies already suggest that adipocytokines have functions in neuronal regulation. Apparently, this will be the first epidemiological study to provide evidence on the association of maternal adipocytokines with the development infant's cognitive status and maternal mental health in the first year postpartum. In this moment, an observational study that shows these possible associations will generate evidence, raise hypotheses and encourage more studies on this topic.

It is hoped that the results will serve as a stimulus for breastfeeding, in addition to to help understand and prevent developmental problems in childhood and throughout the life cycle. In the future, investigation of factors associated with concentrations of adipocytokines in breast milk and interventions that can modulate These concentrations may give rise to new recommendations for postpartum women, generating impact on children's learning capacity and, consequently, improves their social, emotional and cognitive well-being.

Additionally, considering the need for more evidence on the association between adiponectin and depression, the lack of studies on the topic in postpartum women and the impact of depression on maternal and child health, the assessment of the association between

Machine Translated by Google

adipocytokines and mental health in postpartum women is a way to search for new strategies to prevent and treat postpartum depression.

Therefore, the project collaborates with the production and dissemination of knowledge, contributes to the promotion of women's and children's health and quality of life of the Brazilian population and offers subsidies for the execution and formulation of policies national public institutions, being articulated with the institutional plan of Fiocruz and the IFF.

6.1 Expected products

i. Human resources training: 1 scientific initiation work; 2 dissertations master's degree; 1 doctoral thesis;

ii. Production and dissemination of knowledge: 4 articles published in magazines international; 4 abstracts presented at national and international;

7. Goals (definition in quantitative terms) and evaluation criteria

**Goals**	**Rating criteria**
Inclusion of 95 participants	Signing the Consent Form Free and Informed (TCLE) – During the execution of the study.
Contribution to 2 master's theses and 1 doctoral thesis.	Publication in the Institutional Repository from Fiocruz. Available in: https://www.arca.fiocruz.br
Preparation of an executive summary of the impact from the project.	Production of final report – At the end of the study.
Submission of four scientific articles to broad 4 articles diffusion in society (national and International).	running at the end of 24 project months

Machine Translated by Google

Sending results to at least two scientific events.	11th Brazilian Congress of Epidemiology, Fortaleza/ CE, 2020<br>DOHaD Society World Congress, Vancouver/ Canada, 2022

8. Location

Nursing mothers and their newborn children in the IFF maternity ward will be captured in the premises of the Human Milk Bank. Monitoring will take place at the Milk Bank Human (milk collection), in the pediatrics service (child development assessment) and in the Nutrition and Metabolism Laboratory (evaluation of body composition, collection blood test and application of questionnaires).

9. Target audience of the Project, direct and indirect beneficiaries and prescribe actions of interaction between researchers and target audience

The target audience for this project will be postpartum women and their children born in IFF maternity ward. In order to enhance the recruitment of participants, folders distributed in the rooming-in (bedside), for patients who meet the eligibility criteria. The brochures will highlight the possibility of monitoring the baby's body composition and development over the next year and invite them to talk to the team during their childcare at HMB **(Annex** 2).

It is hoped that the results will serve as a stimulus for breastfeeding, in addition to to help understand and prevent developmental problems in childhood and throughout the life cycle. In the future, investigation of factors associated with concentrations of adipocytokines in breast milk and interventions that can modulate These concentrations may give rise to new recommendations for postpartum women, generating impact on children's learning capacity and, consequently, improves their social, emotional and cognitive well-being.

Additionally, considering the need for more evidence on the association between adiponectin and depression, the lack of studies on the topic in postpartum women

Machine Translated by Google

and the impact of depression on maternal and child health, the assessment of the association between adipocytokines and mental health in postpartum women is a way to search for new strategies to prevent and treat postpartum depression.

Interaction between researchers and the target audience will take place throughout the course of the project by reading the Free and Informed Consent Form, carrying out interviews and exams and whenever participants need explanations additional. Furthermore, the results of this study will be disseminated by through workshops for women who have recently given birth to users of the Human Milk Bank, as well as for the employees involved.

10. Detail the continuing education, training and qualification program for qualified human resources

All members of this project will be trained and qualified to carry out conducting interviews and applying the EPDS scale.

- Interview: practical training will be carried out with the group of researchers who will conduct interviews to observe how they are answering the questions and notes on the questionnaire in order to avoid errors when asking questions with the main objective of avoiding the introduction of information bias.
- Body composition assessment: an IFF research fellow, trained and trained in the use of the BOD POD and PEA POD is responsible for this assessment. If If necessary, other team members will be appropriately trained.
- Assessment of the neurodevelopment of the newborn: a team will be part of the psychologist trained and qualified to use the Bayley-III scale.
- After all this training, a pilot study will be carried out to evaluate again the questionnaires, the research flowchart, the performance of the interviewers to the use of instruments.

11. Budget justified

Project duration: 24 months

Table 1. Budget

Description	Amount	Value unitary (R$)	Amount (R$)
**COSTING**			
**Office supplies** (papers, folders, cartridges, pens, notebooks, etc.)	1	2,000.00	2,000.00
**Material for measuring cytokines**			
ELISA KIT for adiponectin measurement	5	2,670.00	13,350.00
ELISA KIT for leptin measurement	5	2,670.00	13,350.00
**Material for collecting samples**			
Cryotubes for sample storage (pack of 1000)	two	318.00	636.00
Cryotube storage boxes	14	7.00	98.00
Universal milk collection collector (pack of 10)	38	5.50	209.00
Disposable needles (box of 100)	3	30.00	90.00
Disposable syringes (box of 100)	3	70.00	210.00
Disposable gloves (box of 100)	6	24.00	144.00
Roll cotton	5	15.00	75.00
EDTA tube for collection (box of 100)	3	47.00	141.00
Garrote	3	16.00	48.00
SCALP (box of 100)	3	27.00	81.00
**Assessment of child development**			
Bayley-III Scale Forms	190	5.00	950.00
**Publication of articles**			
Revisions and translations	1	1,000.00	1,000.00
**Reimbursement of participants**	380	40.00	15,200.00
**Subtotal 1 (Cost)**			47,582.00
SCHOLARSHIPS			
**Middle level**	18	1,200.00	21,600.00
**Higher level**	12	3,000.00	36,000.00
**Subtotal 2 (Scholarships)**			57,600.00
**TOTAL**			

The budget foresees spending on office supplies for printing forms, document storage and activity records. Is being funds were requested to purchase the necessary materials for blood collection and

Machine Translated by Google

To measure cytokines, commercial ELISA kits will be purchased. All material from The laboratory required for this analysis is available at the Institution.

The cost of printing the Bayley-III Scale forms was budgeted, which will be used to assess child development.

Two scholarships are being requested. One will be allocated to a professional middle level who will take blood samples. The second will be aimed at a professional higher level, preferably with experience in the research area, to carry out the field coordination.

R$15,200 reais will be reserved to reimburse expenses arising from the research for the participant and her companion. Reimbursement will be made in all evaluations in which the participant comes to IFF exclusively for the project. That way, Patients external to the IFF will receive reimbursement for all assessments.

The basic infrastructure (living room, minimum furniture consisting of tables and chairs, and closet) and equipment will be provided by the National Institute of Women's Health, of Children and Adolescents, available for use: Bayley- III manipulatives kit, BOD-POD, computers and printers.

Machine Translated by Google

Timeline

Data collection will begin in September 2022, with an estimated duration of 27 months, 15 months of which will involve attracting participants.

Table 2. Schedule

**ACTIVITIES**	**Period**	
	**Start**	**Termination**
**Literature review**	January 2022	July 2025
**Interviewer training**	January 2022	August 2022
**Inclusion of participants**	September 2022	December 2023
**Data collect**	September 2022	December 2024
**Input, monitoring and preliminary analysis** September 2022		December 2024
**Laboratory analyzes of blood and milk samples (T0)**	January 2024	February 2024
**Laboratory analyzes of milk samples (T1)** March 2024		April 2024
**Laboratory analyzes of blood samples and milk (T2)**	July 2024	August 2024
**Laboratory analyzes of blood and milk samples (T3)**	January 2025	February 2025
**Scientific sessions to discuss results**	March 2023	March 2025
**Preparation of scientific articles**	December 2023	July 2025
**Data analysis**	December 2023	July 2025
**Preparation of the final report and accountability**	July 2024	July 2025
**Return of body composition and child development results to participants**	September 2022	December 2024
**Return of the study results report to participants**	July 2024	July 2025

Machine Translated by Google

Bibliographic references

Alder, Judith, Nadine Fink, Johannes Bitzer, Irene Hösli, and Wolfgang Holzgreve. 2007. "Depression and Anxiety During Pregnancy: A Risk Factor for Obstetric, Fetal and Neonatal Outcome? A Critical Review of the Literature". *The Journal of Maternal-Fetal & Neonatal Medicine: The Official Journal of the European Association of Perinatal Medicine, the Federation of Asia and Oceania Perinatal Societies, the International Society of Perinatal Obstetricians* 20 (3): 189–209. https://doi.org/ 10.1080/14767050701209560.

Baldisserotto, Márcia Leonardi, Mariza Miranda Theme-Filha, Rosane Harter Griep, John Oates, Joel Renó Junior, and Juliana Pires Cavalsan. 2018. "Transcultural Adaptation to the Brazilian Portuguese of the *Postpartum Bonding Questionnaire* for Assessing the Postpartum Bond between Mother and Baby". *Public Health Notebooks* 34 (August). https://doi.org/10.1590/0102-311X00170717.

Ballard, Olivia, and Ardythe L. Morrow. 2013. "Human Milk Composition: Nutrients and Bioactive Factors." *Pediatric clinics of North America* 60 (1): 49–74. https://doi.org/10.1016/j.pcl.2012.10.002.

Bertolazi, Alessandra Naimaier, Simone Chaves Fagondes, Leonardo Santos Hoff, Eduardo Giacomolli Dartora, Ilsis Cristine da Silva Miozzo, Maria Emília Ferreira de Barba, and Sérgio Saldanha Menna Barreto. 2011. "Validation of the Brazilian Portuguese Version of the Pittsburgh Sleep Quality Index". *Sleep Medicine* 12 (1): 70–75. https://doi.org/10.1016/j.sleep.2010.04.020.

Bloemer, Jenna, Priyanka D. Pinky, Manoj Govindarajulu, Hao Hong, Robert Judd, Rajesh H. Amin, Timothy Moore, Muralikrishnan Dhanasekaran, Miranda N. Reed, and Vishnu Suppiramaniam. 2018. *“Role* of Adiponectin in Central Nervous System *Disorders.” Neural Plasticity* 2018: 4593530. https://doi.org/10.1155/2018/4593530.

Bolfarine, Heleno, and Wilton de Oliveira Bussab. 2005. *Sampling Elements.*

Boyce, Philip, and Anthea Hickey. 2005. *“Psychosocial* Risk Factors to Major Depression after *Childbirth.” Social Psychiatry and Psychiatric Epidemiology* 40 (8): 605–12. https://doi.org/10.1007/s00127-005-0931-0.

Bravi, Francesca, Frank Wiens, Adriano Decarli, Alessia Dal Pont, Carlo Agostoni, and Monica Ferraroni. 2016. *“Impact* of Maternal Nutrition on Breast-Milk Composition: A Systematic *Review.” The American Journal of Clinical Nutrition* 104 (3): 646–62. https://doi.org/10.3945/ajcn.115.120881.

Buist, A., D. Westley, and C. Hill. 1999. *“Antenatal* Prevention of Postnatal *Depression.” Archives of Women's Mental Health* 1 (4): 167–73. https://doi.org/10.1007/s007370050024.

Camargos, Ana Cristina R., Vanessa A. Mendonça, Katherine SC Oliveira, Camila Alves de Andrade, Hércules Ribeiro Leite, Sueli Ferreira da Fonseca, Erica Leandro Marciano Vieira, Antônio Lúcio Teixeira Júnior, and Ana Cristina Rodrigues Lacerda. 2017. *“Association* between Obesity-Related Biomarkers and Cognitive and Motor Development in *Infants.” Behavioral Brain Research* 325 (Pt A): 12–16. https://doi.org/10.1016/j.bbr.2017.02.030.

Çatlÿ, Gönül, Nihal Olgaç Dündar, and Bumin Nuri Dündar. 2014. *“Adipokines* in Breast Milk: An *Update.” Journal of Clinical Research in Pediatric Endocrinology* 6(4): 192–201. https://doi.org/10.4274/Jcrpe.1531.

Codoñer-Franch, Pilar, María T. Hernández-Aguilar, Almudena Navarro-Ruiz, Ana B. López-Jaén, Cintia Borja-Herrero, and Victoria Valls-Bellés. 2013. "Diet

Machine Translated by Google

Supplementation during Early Lactation with Non-Alcoholic Beer Increases the Antioxidant Properties of Breastmilk and Decreases the Oxidative Damage in Breastfeeding Mothers." *Breastfeeding Medicine: The Official Journal of the Academy of Breastfeeding Medicine* 8 (April): 164–69. https:// doi.org/10.1089/bfm.2012.0059.

Costa, Caroline dos Santos, Franciane Rocha de Faria, Kamila Tiemann Gabe, Isabela Fleury Sattamini, Neha Khandpur, Fernanda Helena Morocco Leite, Eurídice Martínez Steele, Maria Laura da Costa Louzada, Renata Bertazzi Levy, and Carlos Augusto Monteiro. 2021. "Nova Score for consumption of ultra-processed foods: description and performance assessment in Brazil". *Public Health Magazine* 55 (April). https://doi.org/10.11606/s1518-8787.2021055003588.

Cox, JL, JM Holden, and R Sagovsky. 1987. *"Detection* of Postnatal Depression. Development of the 10-Item Edinburgh Postnatal Depression Scale". *The British Journal of Psychiatry: The Journal of Mental Science* 150 (June): 782–86.

Fields, David A., Paul B. Higgins, and Gary R. Hunter. 2004. *"Assessment* of Body Composition by Air-Displacement Plethysmography: Influence of Body Temperature and *Moisture." Dynamic Medicine: DM* 3 (1): 3. https://doi.org/10.1186/1476-5918-3-3.

Filgueiras, Alberto, Pedro Pires, Silvia Maissonette, and J. Landeira-Fernandez. 2013. "Psychometric Properties of the Brazilian-Adapted Version of the Ages and Stages Questionnaire in Public Child Daycare Centers". *Early Human Development* 89(8):561–76. https://doi.org/10.1016/j.earlhumdev.2013.02.005.

Garza, Jacob C., Ming Guo, Wei Zhang, and Xin-Yun Lu. 2008. *"Leptin* Increases Adult Hippocampal Neurogenesis in Vivo and in *Vitro." The Journal of Biological Chemistry* 283 (26): 18238–47. https://doi.org/10.1074/jbc.M800053200.

Gomes-Oliveira, Marcio Henrique, Clarice Gorenstein, Francisco Lotufo Neto, Laura Helena Andrade, and Yuan Pang Wang. 2012. "Validation of the Brazilian Portuguese Version of the Beck Depression Inventory-II in a Community Sample". *Brazilian Journal of Psychiatry* 34 (December): 389–94. https://doi.org/10.1016/j.rbp.2012.03.005.

Gorska-Ciebiada, Malgorzata, Malgorzata Saryusz-Wolska, Anna Borkowska, Maciej Ciebiada, and Jerzy Loba. 2016. "Adiponectin, leptin and IL-1 ÿ in elderly diabetic patients with mild cognitive impairment." *Metabolic Brain Disease* 31: 257–66. https:// doi.org/10.1007/s11011-015-9739-0.

Grantham-McGregor, Sally, Yin Bun Cheung, Santiago Cueto, Paul Glewwe, Linda Richter, and Barbara Strupp. 2007. *"Developmental* Potential in the First 5 Years for Children in Developing *Countries." The Lancet* 369 (9555): 60–70. https://doi.org/10.1016/S0140-6736(07)60032-4.

Gross, Rachel S, Nerissa K Velazco, Rahil D Briggs, and Andrew D Racine. 2013. "Maternal Depressive Symptoms and Child Obesity in Low-Income Urban Families". *Academic Pediatrics* 13 (4): 356–63. https://doi.org/10.1016/j.acap.2013.04.002.

Gunstad, John, Mary Beth Spitznagel, Therese A. Keary, Ellen Glickman, Thomas Alexander, Jessica Karrer, Kelly Stanek, Lynn Reese, and Judi Juvancic-Heltzel. 2008. *"Serum* Leptin Levels Are Associated with Cognitive Function in Older *Adults." Brain Research* 1230 (September): 233–36. https://doi.org/10.1016/j.brainres.2008.07.045.

Guo, M., C. Li, Y. Lei, S. Xu, D. Zhao, and X.-Y. Lu. 2017. "Role of the Adipose PPARÿ-Adiponectin Axis in Susceptibility to Stress and Depression/Anxiety-Related

Machine Translated by Google

Behaviors". *Molecular Psychiatry* 22 (7): 1056–68. https://doi.org/10.1038/mp.2016.225.

Harvey, Jenni. 2007. "Leptin regulation of neuronal excitability and cognitive function." *Current Opinion in Pharmacology* 7(6–3): 643–47. https://doi.org/10.1016/j.coph.2007.10.006.

Hibbeln, Joseph R. 2002. "Seafood consumption, the DHA content of mothers' milk and prevalence rates of postpartum depression: a cross-national, ecological analysis." *Journal of affective disorders* 69 (1): 15–30.

Holden, Karen F., Karla Lindquist, Frances A. Tylavsky, Caterina Rosano, Tamara B. Harris, and Kristine Yaffe. 2009. "Serum leptin level and cognition in the elderly: Findings from the Health ABC Study." *Neurobiology of aging* 30 (9): 1483–89. https://doi.org/10.1016/j.neurobiolaging.2007.11.024.

Jensen, Sarah KG, Iroise Dumontheil, and Edward D Barker. 2013. *"Developmental* Inter-Relations between Early Maternal Depression, Contextual Risks, and Interpersonal Stress, and Their Effect on Later Child Cognitive *Functioning." Depression and Anxiety,* September. https://doi.org/10.1002/da.22147.

Lanza di Scalea, Teresa, and Katherine L Wisner. 2009. *"Antidepressant* Medication Use During *Breastfeeding." Clinical Obstetrics and Gynecology* 52 (3): 483–97. https://doi.org/10.1097/GRF.0b013e3181b52bd6.

Lee, Thomas Ho-yin, Kenneth King-yip Cheng, Ruby Lai-chong Hoo, Parco Ming-fai Siu, and Suk-yu Yau. 2019. *"The* Novel Perspectives of Adipokines on Brain *Health." International Journal of Molecular Sciences* 20 (22): 5638. https://doi.org/10.3390/ijms20225638.

Li, Nan, Tye E. Arbuckle, Gina Muckle, Bruce P. Lanphear, Michel Boivin, Aimin Chen, Linda Dodds, et al. 2019. *"Associations* of Cord Blood Leptin and Adiponectin with *Children's* Cognitive *Abilities." Psychoneuroendocrinology* 99: 257–64. https://doi.org/10.1016/j.psyneuen.2018.10.021.

Liu, J., M. Guo, D. Zhang, S.-Y. Cheng, M. Liu, J. Ding, P. E. Scherer, F. Liu, and X.-Y. Lu. 2012. "Adiponectin is critical in determining susceptibility to depressive behaviors and has antidepressant-like activity." *Proceedings of the National Academy of Sciences* 109 (30): 12248–53. https://doi.org/10.1073/pnas.1202835109.

Mello, Marco Túlio de, Ana R. Dâmaso, Hanna Karen M. Antunes, Kãli O. Siqueira, Marise Lazaretti Castro, Sheila V. Bertolino, Sérgio G. Stella, and Sérgio Tufik. 2005. "Assessment of body composition in obese adolescents: the use of two different methods." *Brazilian Journal of Sports Medicine* 11 (5): 267–70. https://doi.org/10.1590/S1517-86922005000500004.

Oates, Margaret. 2003. *"Suicide:* The Leading Cause of Maternal *Death." The British Journal of Psychiatry* 183 (4): 279–81. https://doi.org/10.1192/bjp.183.4.279.

O'Malley, Dervla, Neil MacDonald, Sarah Mizielinska, Christopher N. Connolly, Andrew J. Irving, and Jenni Harvey. 2007. *"Leptin* promotes rapid dynamic changes in hippocampal dendritic *morphology." Molecular and Cellular Neurosciences* 35(4):559–72. https://doi.org/10.1016/j.mcn.2007.05.001.

Otto, SJ, RHM de Groot, and G Hornstra. 2003. "Increased risk of postpartum depressive symptoms is associated with slower normalization after pregnancy of the functional docosahexaenoic acid status". *Prostaglandins, Leukotrienes and Essential Fatty Acids* 69 (4): 237–43. https://doi.org/10.1016/S0952-3278(03)00090-5.

Rebelo, Fernanda, Dayana R Farias, Claudio J Struchiner, and Gilberto Kac. 2016. "Plasma Adiponectin and Depressive Symptoms During Pregnancy and the

Machine Translated by Google

Postpartum Period: A Prospective Cohort Study". *Journal of Affective Disorders* 194 (April): 171–79. https://doi.org/10.1016/j.jad.2016.01.012.

Rebelo, Fernanda, Thatiana de Jesus Pereira Pinto, Ana Beatriz Franco-Sena, Jaqueline Lepsch, Camila Benaim, Claudio José Struchiner, and Gilberto Kac. 2015. "Plasma Adiponectin Is Inversely Associated with Antenatal Anxiety: Results from a Brazilian Cohort." *Psychoneuroendocrinology* 51 (January): 92–100. https://doi.org/10.1016/j.psyneuen.2014.09.015.

Santos, Iná S., Alicia Matijasevich, Beatriz Franck Tavares, Aluísio JD Barros, Iara Picinini Botelho, Catherine Lapolli, Pedro Vieira da Silva Magalhães, Ana Paula Pereira Neto Barbosa, and Fernando C. Barros. 2007. "Validation of the Edinburgh Postnatal Depression Scale (EPDS) in a Sample of Mothers from the 2004 Pelotas Birth Cohort Study." *Cadernos De Saúde Pública* 23 (11): 2577–88.

Savino, Francesco, and Stefania A. Liguori. 2008. "Update on Breast Milk Hormones: Leptin, Ghrelin and Adiponectin." *Clinical Nutrition (Edinburgh, Scotland)* 27 (1): 42–47. https://doi.org/10.1016/j.clnu.2007.06.006.

Sit, Dorothy, James M. Perel, Stephen R. Wisniewski, Joseph C. Helsel, James F. Luther, and Katherine L. Wisner. 2011. *“Mother*-Infant Antidepressant Concentrations, Maternal Depression, and Perinatal *Events.” The Journal of Clinical Psychiatry* 72 (7): 994–1001. https://doi.org/10.4088/JCP.10m06461.

Textor, Johannes, Juliane Hardt, and Sven Knüppel. 2011. "DAGitty: A Graphical Tool for Analyzing Causal Diagrams." *Epidemiology (Cambridge, Mass.)* 22 (5): 745. https://doi.org/10.1097/EDE.0b013e318225c2be.

Venancio, Sonia I., Maria ML Escuder, Sílvia RDM Saldiva, and Elsa RJ Giugliani. 2010. "Breastfeeding practices in the Brazilian capital cities and the Federal District: current status and advances". *Journal of Pediatrics* 86 (4): 317–24. https://doi.org/10.1590/S0021-75572010000400012.

Villar, José, Leila Cheikh Ismail, Cesar G. Victora, Eric O. Ohuma, Enrico Bertino, Doug G. Altman, Ann Lambert, et al. 2014. *“International* Standards for Newborn Weight, Length, and Head Circumference by Gestational Age and Sex: The Newborn Cross-Sectional Study of the INTERGROWTH-21st *Project.” Lancet (London, England)* 384 (9946): 857–68. https://doi.org/10.1016/S0140-6736(14)60932-6.

Yildiz, Gazi, Mehmet Baki Senturk, Pinar Yildiz, Yusuf Cakmak, Mehmet Sukru Budak, and Erbil Cakar. 2017. *“Serum* Serotonin, Leptin, and Adiponectin Changes in Women with Postpartum Depression: Controlled *Study.” Archives of Gynecology and Obstetrics* 295 (4): 853–58. https://doi.org/10.1007/s00404-017-4313-0.

Young, B. E., C. Levek, R. M. Reynolds, M. C. Rudolph, P. MacLean, T. L. Hernandez, JE Friedman, and NF Krebs. 2018. *“Bioactive* Components in Human Milk Are Differentially Associated with Rates of Lean and Fat Mass Deposition in Infants of Mothers with Normal vs. Elevated *BMI.” Pediatric Obesity* 13 (10): 598–606. https://doi.org/10.1111/ijpo.12394.

Zhang, D., M. Guo, W. Zhang, and X.-Y. Lu. 2011. "Adiponectin Stimulates Proliferation of Adult Hippocampal Neural Stem/Progenitor Cells through Activation of P38 Mitogen-Activated Protein Kinase (P38MAPK)/Glycogen Synthase Kinase 3 (GSK-3)/ -Catenin Signaling Cascade." *Journal of Biological Chemistry* 286(52):44913–20. https://doi.org/10.1074/jbc.M111.310052.

Machine Translated by Google

Annex 1 – Edinburgh Postnatal Depression Scale (EPDS)

Edinburgh Quiz		
You've had a baby and we'd like to know how you're feeling. To do this, I ask you to answer the option that comes closest to how you felt IN THE LAST SEVEN DAYS, and not just how you feel today.		
1	I have been able to laugh and find things funny.	0] As I always did.<br>[1] Not as much as before.<br>[2] Without a doubt, less than before.<br>[3] No way.
two	I have been thinking about the future with joy.	[0] Yes, as usual.<br>[1] A little less than usual.<br>[2] Much less than usual.<br>[3] Practically no.
3	I have been blaming myself for no reason when things go wrong.	[0] No, not at all.<br>[1] Rarely.<br>[2] Yes, sometimes.<br>[3] Yes, very often.
4	I have been anxious or worried for no good reason.	[3] Yes, very followed.<br>[2] Yes, sometimes.<br>[1] From time to time.<br>[0] No, not at all.
5	I have been feeling scared or panicked for no good reason.	[3] Yes, very followed.<br>[2] Yes, sometimes.<br>[1] Rarely.<br>[0] No, not at all.
6	I have felt overwhelmed by the tasks and events of my day-to-day life	[3] Yes. Most of the time I can't deal with them well.<br>[2] Yes. Sometimes I can't cope as well as I used to.<br>[1] No. Most of the time I can deal with them well.<br>[0] No. I can handle them as well as ever.
7	I've been feeling so unfortunate that I have been having difficulty sleeping.	[3] Yes, most of the time.<br>[2] Yes, sometimes.<br>[1] Rarely.<br>[0] No, not once.
8	I have been feeling sad or really bad.	[3] Yes, most of the time.<br>[2] Yes, often.<br>[1] Rarely.<br>[0] No, not at all.
9	I have been feeling so sad that I have been crying.	[3] Yes, most of the time.<br>[2] Yes, often.<br>[1] Only once in a while.<br>[0] No, never.
10	I've been thinking about doing something against myself same.	[3] Yes, often.]<br>[2 Sometimes.<br>[1] Rarely.<br>[0 [ ] Never.

Machine Translated by Google

Annex 2 – Disclosure folder

Já imaginou poder acompanhar a sua composição corporal e o desenvolvimento cognitivo e motor do seu bebê durante o próximo ano com as melhores avaliações disponíveis?

Se você tem de 20 a 45 anos de idade e teve parto a termo (37 semanas ou mais), converse com uma das pesquisadoras do Projeto APPLE na sua visita ao banco de leite.

Traga uma roupa justa (top e short) para avaliação da sua composição corporal.
