# Supplementary material for: Plasma and breast milk adipokines in women across the first year postpartum and their association with maternal depressive symptoms and infant neurodevelopment: Protocol for the APPLE prospective cohort study
Source: PLoS One. 2024 Oct 25;19(10):e0310847. doi: 10.1371/journal.pone.0310847 (PMC11508165; doi:10.1371/journal.pone.0310847)
Supplement: Attachment — Submitted filename: Response to Reviewers.docx [file pone.0310847.s004.docx]

**Rebuttal Letter**

Dear Editor and Reviewer,

Thank you for your time and effort in evaluating our manuscript. We are very grateful for the comments and suggestions, which for sure contributed to enhance the quality of our study.

The answers and considerations for each comment are listed below. The comments are in black and the answers are in red. All the changes are marked in yellow in the “Revised Manuscript with Track Changes” file.

Journal requirements:

1. When submitting your revision, we need you to address these additional requirements.

Please ensure that your manuscript meets PLOS ONE's style requirements, including those for file naming. The PLOS ONE style templates can be found at 
<https://journals.plos.org/plosone/s/file?id=wjVg/PLOSOne_formatting_sample_main_body.pdf> and 
<https://journals.plos.org/plosone/s/file?id=ba62/PLOSOne_formatting_sample_title_authors_affiliations.pdf>.

We revised the style according to PLOS ONE’s requirements and made the necessary corrections.

2. We note that you have selected “Clinical Trial” as your article type. PLOS ONE requires that all clinical trials are registered in an appropriate registry (the WHO list of approved registries is at <https://www.who.int/clinical-trials-registry-platform/network/primary-registries>" <https://www.who.int/clinical-trials-registry-platform/network/primary-registries> and more information on trial registration is at <http://www.icmje.org/about-icmje/faqs/clinical-trials-registration/>). Please state the name of the registry and the registration number (e.g. ISRCTN or ClinicalTrials.gov) in the submission data and on the title page of your manuscript. a) Please provide the complete date range for participant recruitment and follow-up in the methods section of your manuscript. b) If you have not yet registered your trial in an appropriate registry, we now require you to do so and will need confirmation of the trial registry number before we can pass your paper to the next stage of review. Please include in the Methods section of your paper your reasons for not registering this study before enrolment of participants started. Please confirm that all related trials are registered by stating: “The authors confirm that all ongoing and related trials for this drug/intervention are registered”. Please see <http://journals.plos.org/plosone/s/submission-guidelines#loc-clinical-trials> for our policies on clinical trials.

We did not select 'Clinical Trial' as the article type. On the submission page, we chose 'Study Protocol.' Our study is not a clinical trial but an observational cohort study. Nevertheless, the study is registered with the Brazilian Clinical Trials Registry (ReBec) under the number RBR-9hcby8c, as mentioned in the title page and methods section. The full date range for participant recruitment and follow-up has been provided and updated on page 8, line 229.

3. Thank you for stating the following financial disclosure: 
 [The project is being funded by the Research Support Program (APQ-1) of the Rio de Janeiro State Research Support Foundation (FAPERJ Proc. No. 211.557/2021)].  
Please state what role the funders took in the study.  If the funders had no role, please state: ""The funders had no role in study design, data collection and analysis, decision to publish, or preparation of the manuscript."" 
If this statement is not correct you must amend it as needed. 
Please include this amended Role of Funder statement in your cover letter; we will change the online submission form on your behalf.

As stated in the manuscript file and now added to the cover letter:

‘The project is being funded by the Research Support Program (APQ-1) of the Rio de Janeiro State Research Support Foundation (FAPERJ Proc. No. 211.557/2021). Participant reimbursement is being provided with support from the Research Productivity Fellowship from the National Council for Scientific and Technological Development (CNPq) under the name of AEN. COM, LGR and TMT receive scholarships from Coordination for the Improvement of Higher Education Personnel (CAPES). MTNR receive scholarship from CNPq. The funders had no role in study design, data collection and analysis, decision to publish, or preparation of the manuscript.’

4. We note that you have indicated that there are restrictions to data sharing for this study. PLOS only allows data to be available upon request if there are legal or ethical restrictions on sharing data publicly. For more information on unacceptable data access restrictions, please see <http://journals.plos.org/plosone/s/data-availability#loc-unacceptable-data-access-restrictions>.

The data availability statement has been updated to more accurately reflect the current status of this article.

New text: ‘This article does not report any data, so the data availability policy is not applicable. Upon completion of the APPLE project, the final database containing de-identified research data will be deposited in Arca Dados (https://arcadados.fiocruz.br), the official repository of Fundação Oswaldo Cruz.’

Before we proceed with your manuscript, please address the following prompts:

a) If there are ethical or legal restrictions on sharing a de-identified data set, please explain them in detail (e.g., data contain potentially identifying or sensitive patient information, data are owned by a third-party organization, etc.) and who has imposed them (e.g., a Research Ethics Committee or Institutional Review Board, etc.). Please also provide contact information for a data access committee, ethics committee, or other institutional body to which data requests may be sent.

This article does not report any data, so the data availability policy is not applicable. Upon completion of the APPLE project, the final database containing de-identified research data will be deposited in Arca Dados (https://arcadados.fiocruz.br), the official repository of Fundação Oswaldo Cruz.

b) If there are no restrictions, please upload the minimal anonymized data set necessary to replicate your study findings to a stable, public repository and provide us with the relevant URLs, DOIs, or accession numbers. For a list of recommended repositories, please see
<https://journals.plos.org/plosone/s/recommended-repositories>. You also have the option of uploading the data as Supporting Information files, but we would recommend depositing data directly to a data repository if possible.

We will update your Data Availability statement on your behalf to reflect the information you provide.

This article does not report any data, so the data availability policy is not applicable. Upon completion of the APPLE project, the final database containing de-identified research data will be deposited in Arca Dados (https://arcadados.fiocruz.br), the official repository of Fundação Oswaldo Cruz.

5. Please include captions for your Supporting Information files at the end of your manuscript, and update any in-text citations to match accordingly. Please see our Supporting Information guidelines for more information: <http://journals.plos.org/plosone/s/supporting-information>.

The captions for the Supporting Information files have been added to the end of the manuscript. Additionally, we included a sentence at the end of the Methods section to provide an in-text citation for the Supporting Information.

Additional Editor Comments:

1. Please provide a valid rationale for the proposed study by clearly identify and justify research questions

A paragraph was inserted in the end of the introduction to clearly identify and justify research questions.

2. Descriptions of methods and materials in the protocol should be reported in sufficient detail

We did a careful review of the methods section, and we are convicted that all details are described.

3. Discussion should be more elaborated, including implication to clinical practice

A more detailed explanation of the implications of the results for clinical practice has been included in the Discussion section.

4. Please pay more attention to manuscript writing guidelines and grammar
Thank you, we did a careful review of writing and grammar.
_________________________________________________________________________

Reviewer #1: Comments

Line 164 , Line 77, Line 358, Line 366, Line 373. the term 'waves' could be omitted.
Thank you for the suggestion. The word ‘waves’ was removed from the indicated sentences.

Line 374, the language version of Nova24h and Line 419, the amount of reimbursement is to be stated.
The required information has been entered in the respective lines. The reimbursement amount varies among participants. Each participant received the total amount they spent due to their participation in the project.

Line 426-427, the information provided is unclear. More information is to be provided e.g at which time point measurement and the values. In reference 53, 13.6 is referring to the age interval (months) 6.
Line -430, E= difference considered significant, δ=SD, Zα/2 =1.96 is to be denoted.
Line 430, the sample size formula presentation n=[Zα/2.δ/E]²) requires revision
Thank you for the careful revision. The formula presentation indeed needed revision, and additional information was necessary to enhance readers' understanding. We have made the required modifications.

Line 440, proper citation for STATA software is to be provided.
The proper citation was provided.

Line 447-448, the type of adipokines e.g. adiponectin and leptin are to be stated.

Thank you, the suggestion was accepted.

Preliminary results are to be omitted as the focus of the paper is describing the methodology. If the authors believe the preliminary results are important, the author may consider including them as supplementary files if allowable by the journal.

The preliminary results were removed from the manuscript.
